# A Sustainable Bio‐Based Epoxy Thermoset as a High‐Performance Alternative to BPA‐Based Resins

**DOI:** 10.1002/advs.76659

**Published:** 2026-07-20

**Authors:** Jiyun Qi, Xinyi Hui, Xipeng Zhang, Jia‐Long Wen, Tong‐Qi Yuan

**Affiliations:** ^1^ State Key Laboratory of Efficient Production of Forest Resources Beijing Forestry University Beijing China; ^2^ Beijing Key Laboratory of Lignocellulosic Chemistry Beijing Forestry University Beijing China

**Keywords:** bio‐based epoxy resin, dynamic ester networks, lignin, structural adhesives

## Abstract

Epoxy resins are indispensable to structural composites, coatings, and adhesives. However, conventional bisphenol A (BPA)‐based epoxy resins are limited by toxicity concerns, intrinsic brittleness, poor end‐of‐life recyclability, and a high carbon footprint. Here, a rigid–flexible bio‐based network design is developed by coupling epoxidized soybean oil (ESO), lignin, and malic acid through maleic anhydride bridging, which simultaneously improves component compatibility and introduces dynamic ester linkages for pH‐responsive degradation. The resulting thermoset achieved an unusual combination of strength, ductility, and environmental resilience, delivering 22.08 MPa shear strength as a structural adhesive and 31 4.57 MPa tensile strength in glass‐fabric composites, together with an ≈250% increase in flexural strain relative to the BPA epoxy. It further maintains robust performance after exposure to chemically aggressive media and extreme temperatures ranging from −196 to 200°C. Life cycle assessment (LCA) and techno‐economic analysis (TEA) confirmed its environmental friendliness and competitive cost‑effectiveness. This work demonstrates a sustainable design for high‑performance thermoset, offering a viable sustainable alternative for applications such as structural adhesives and wind‑turbine composites, thereby accelerating the transition toward a circular bio‑economy.

## Graphical abstract

A rigid–flexible bio‐based epoxy network integrates soybean oil, lignin, and maleic anhydride to overcome the strength–toughness–sustainability trade‐off of conventional BPA epoxy. The thermoset delivers excellent performance as both a structural adhesive and a matrix for glass‐fabric composites, while enabling pH‐responsive degradation, environmental tolerance, lower environmental impact, and competitive cost.

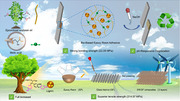

## 1 Introduction

**FIGURE 1:**
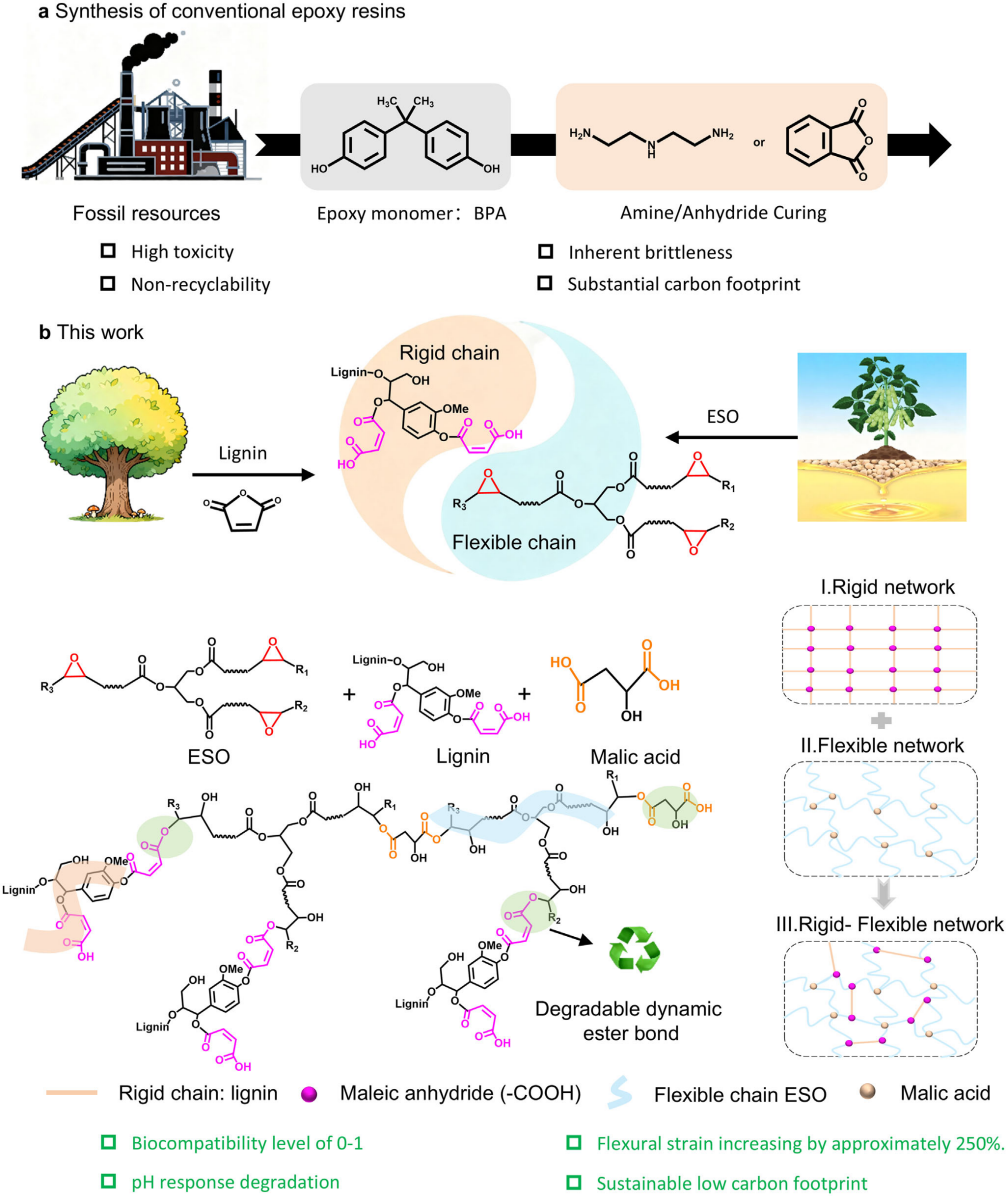
Schematic illustration of the resin synthesis routes. (a) Illustration of the unsustainable status and four critical drawbacks of fossil resource‐based BPA thermosetting resins. (b) The synthesis and pH‐responsive degradation of a fully biomass‐derivable epoxy resin from epoxidized soybean oil, lignin, and malic acid.

Epoxy resin serves as an indispensable structural and functional material in modern industry, with critical applications ranging from wind turbine blades and aerospace composites to electronic encapsulation and high‐performance coatings and adhesives. However, the currently dominant bisphenol A (BPA)‐based epoxy resins face a series of severe challenges, which have significantly hindered their transition toward greener material systems [1, 2]. Specifically, BPA has been confirmed to exhibit endocrine‐disrupting activity, posing a potential threat to ecosystems and human health [3]. Meanwhile, their highly crosslinked network structure resulted in insufficient intrinsic toughness, making them prone to brittle fracture under impact or fatigue loading. Furthermore, the permanent covalent crosslinked networks prevented the cured materials from being degraded in a controllable or stimuli‐responsive manner, thereby increasing end‐of‐life disposal pressure and limiting their sustainable management. Additionally, their synthesis remained entirely dependent on fossil resources, leading to substantial carbon emissions throughout the entire lifecycle from raw materials to final products (Figure 1a) [4]. These limitations have driven growing interest in bio‐based epoxy materials, yet simultaneously achieving high mechanical performance, toughness, and sustainability within a single thermoset platform remains a major challenge.

Among renewable epoxy precursors, epoxidized soybean oil (ESO) is particularly attractive due to its abundance, low cost, and high epoxide functionality, with an annual global production exceeding 250 000 metric tons [5, 6, 7]. Nevertheless, ESO‐derived thermosets generally suffer from insufficient stiffness and strength because of their flexible aliphatic chains and relatively low crosslinking efficiency, which limits their use in demanding structural applications [8]. Lignin, by contrast, is the second‐most abundant terrestrial biopolymer, with an annual production of 50–70 million tons as a byproduct of the paper industry [9]. Lignin offers a rigid aromatic framework that is advantageous for enhancing stiffness, strength, and thermal stability [10]. Its abundant hydroxyl functionalities also provide opportunities for chemical modification and covalent incorporation into epoxy networks. These complementary features suggest that integrating ESO and lignin could provide a promising route toward bio‐based epoxy thermosets with a balanced combination of strength and deformability [7, 11].

Based on the intrinsic material properties of ESO and lignin, we hypothesized that a fully bio‐based, “rigid–flexible” synergistic molecular design synergistic might offer a promising pathway to address these interconnected limitations (Figure 1b). A key obstacle to this strategy, however, lies in the poor compatibility between hydrophobic ESO and highly polar, structurally heterogeneous lignin, which often leads to phase separation and compromised network homogeneity. Addressing this issue requires molecular‐level regulation of lignin polarity, reactivity, and interfacial interactions [12, 13]. Among various approaches, esterification is recognized as an effective method [14, 15, 16]. Here, maleic anhydride is introduced as a multifunctional molecular bridge to esterify lignin [17], thereby improving its compatibility with ESO while simultaneously creating reactive sites for network formation. More importantly, the residual carboxyl groups after esterification and the resulting ester bonds collectively established a pH‐responsive dynamic covalent bond system, endowing the material with reversible cleavage and reassembly capabilities [18]. Malic acid is further incorporated as a bio‐based co‐monomer to assist curing and optimize the crosslinked architecture. On this basis, a rigid–flexible synergistic design is established, in which ESO provides a deformable aliphatic phase, lignin contributes rigid aromatic reinforcement, and maleic anhydride‐bridged interfacial regulation together with malic‐acid‐assisted curing enables the construction of an integrated bio‐based epoxy network.

Beyond molecular design, the practical relevance of such materials depends on whether they can meet the requirements of advanced structural applications. Accordingly, the resulting epoxy thermoset is evaluated not only as a structural adhesive but also as a matrix for glass‐fiber‐reinforced composites, with particular relevance to lightweight and durable composite systems such as wind‐energy structures. To assess the sustainability of this material platform beyond feedstock substitution alone, life cycle assessment (LCA) and techno‐economic analysis (TEA) are further conducted to quantify its environmental and economic profiles. Collectively, this work establishes a viable design strategy for high‐performance bio‐based epoxy thermosets that addresses key limitations of conventional BPA‐based systems.

## 2 Results and Discussion

### 2.1 Green Synthesis of Maleic Anhydride‐Esterified Lignin

Lignin, rich in reactive functional groups such as hydroxyl and carboxyl groups, endows it with high reactivity. ESO, as a biomass‐derived feedstock, contains abundant epoxy groups. Consequently, lignin holds potential as a cross‐linking skeleton for ESO to construct a covalent network. However, the high polarity of lignin results in poor compatibility with ESO. Furthermore, the inherently condensed structure of industrial lignin restricted the accessibility of its reactive sites [19], which frequently resulted in incomplete crosslinking when it is directly utilized as a crosslinker. To overcome these challenges, this study employed maleic anhydride as a multifunctional esterification agent to modify lignin. Comprehensive analysis by FTIR and NMR spectroscopy revealed that maleic anhydride underwent site‐selective esterification with lignin while preserving its aromatic backbone, ultimately yielding a modified lignin with enhanced reactivity and structural integrity (Figures S1–S5 and Table S2).

The maleic anhydride esterification reaction significantly altered the structure and physicochemical properties of lignin by covalently grafting ester groups onto its hydroxyl sites [20]. Following esterification, the molecular weight of lignin increased (Mw from 2029 Da to 2673 Da) with a more uniform distribution (PDI decreased from 2.22 to 2.01) (Figure S6). This structural modification induced a series of changes: the newly introduced ester bonds moderately reduced the thermal stability (Figure S7 and Table S3), while the increased molecular weight and cross‐linked network enhanced mass transfer resistance, thereby slowing the overall pyrolysis rate [21]. Concurrently, the incorporation of ester groups disrupted the original intermolecular hydrogen bonding and π–π stacking interactions [22, 23], leading to the reorganization of lignin from irregular aggregates into smaller and smoother quasi‐spherical particles (Figure S8) [24]. Although the ester groups are polar, the reduction of hydroxyl groups and rearrangement of molecular chains collectively lowered the surface energy [25], markedly improving hydrophobicity (contact angle increased from 64.09° to 90.45°) (Figure S9) and enhancing solubility in various organic solvents (Figure S10) [26]. These changes significantly improved the compatibility and dispersion stability of lignin in organic systems, establishing a foundation for its use as a high‐performance bio‐based curing agent or reinforcing phase in epoxy resin synthesis.

### 2.2 “Rigid–Flexible” Thermoset Network Design

**FIGURE 2:**
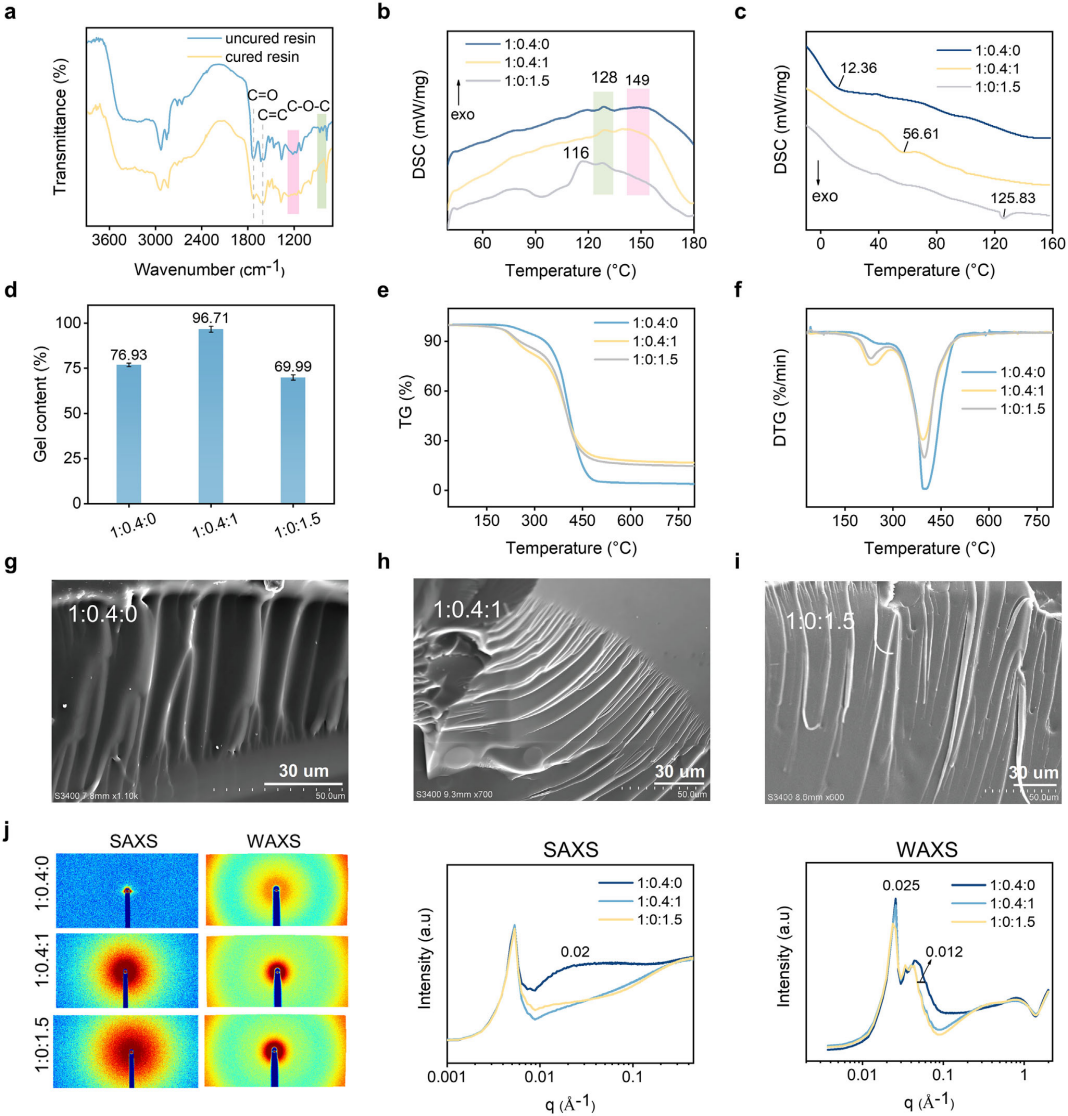
Experimental characterizations of bio‐based epoxy resin. (a) FTIR microscopy images of uncured and cured resins. (b) DSC curves of the resins. (c) The resins exhibited different glass transition temperatures with different contents of lignin. (d) Gel content of the cured networks. (e,f) TG/DTG curves. (g–i) SEM of the liquid‐nitrogen‐fractured resins. (j) SAXS and WAXS scattering images and curves.

A fully bio‐based thermosetting epoxy resin system was constructed by reacting maleic anhydride‐esterified lignin as a multifunctional curing agent with ESO and malic acid. The ternary components synergistically formed a unique topologically crosslinked network, resulting in a novel sustainable material with tunable thermomechanical properties and environmentally friendly characteristics. FTIR spectroscopy revealed the structural evolution of the bio‐based epoxy resin during curing (Figure 2a). The significant attenuation of the ester carbonyl (1735 cm^−^^1^) and C─O stretching vibrations (1210 cm^−^^1^) indicated the concurrent participation of ester bonds and newly formed ether linkages in the construction of the cross‐linked network; the complete disappearance of the characteristic epoxy ring vibrations (821–867 cm^−^^1^) confirmed ring‐opening and successful incorporation into the polymer matrix. From a reaction kinetics perspective, the introduction of esterified lignin significantly reduced the reaction energy barrier [27, 28], accompanied by narrowing of the curing exotherm, indicating accelerated densification of the three‐dimensional network (Figure 2b). This kinetic advantage was directly reflected in the continuous increase in the glass transition temperature (Tg) with lignin content (Figure 2c), suggesting that the rigid aromatic structure restricted chain mobility, while hydroxyl and ester groups acted as cross‐linking sites contributing to a high‐density network. Finally, at the optimal ratio of 1:0.4:1, the gel content reached 96.71% (Figure 2d), further verifying the notable enhancement of network integrity through chemical cross‐linking and catalytic effects.

The bio‑based epoxy resin demonstrated excellent integrated properties, primarily attributed to its unique “rigid‑flexible multiphase structure” formed by the flexible chains of epoxidized soybean oil and the rigid core of lignin. The resin combined good thermal processability (Figure S11) with an improved high‐temperature protective response. The substantially increased char residue, from 3.79% to 16.70% (Figure 2e,f and Table S4), suggested more efficient construction of a stable carbonaceous barrier in the condensed phase. This char‐forming capability, together with the favorable behavior under vertical flame exposure (Figure S12 and Videos S1 and S2), indicated enhanced flame‐retardant performance [29, 30]. It also displayed hydrophobic stability, with the water contact angle of the optimal formulation decreasing by only 9.75° over 300 s (Figure S13). In terms of electrical properties, the breakdown strength was improved by 26.14% compared to conventional systems (Figure S14). Furthermore, the material exhibited intelligent shape memory behavior with temperature‐dependent recovery and pH‐responsive degradability [31], enabling complete hydrolysis under alkaline conditions for controllable life‐cycle management (Figures S15–S17) [32]. These combined characteristics establish the resin as a versatile materials platform integrating high performance, environmental responsiveness, and sustainability, showing promising potential for applications in recyclable composites, smart adhesives, and green electronics.

Through multi‐scale structural characterization, the internal features of the fully bio‐based epoxy resin were clearly elucidated. SEM observations revealed distinct tough‐fracture behaviors among the resin networks (Figure 2g–i and Figure S18). The ESO–malic acid resin displayed abundant oriented fibrillar structures, indicating that the flexible ESO chains dissipated fracture energy through chain slippage, orientation, and necking‐induced plastic deformation. After introducing rigid lignin, the fracture morphology of the ESO–lignin–malic acid ternary resin evolved from tensile fibrillation to fan‐shaped shear‐slip features, suggesting that lignin effectively suppressed straight crack propagation and induced crack deflection, thereby enhancing matrix rigidity while retaining toughness [33]. In contrast, the ESO–lignin resin exhibited both dense fibrillar pulling and rough tearing dimples, reflecting a synergistic energy‐dissipation mechanism involving internal plasticization from flexible ESO segments and lignin‐induced crack deflection. Overall, lignin effectively regulated chain mobility and crack‐propagation pathways within the crosslinked network, enabling a transition from high flexibility toward balanced stiffness and toughness. Small‐angle x‐ray scattering (SAXS) data revealed that this formulation possessed a homogeneous and continuous nanoscale phase structure, as evidenced by a significantly weakened scattering peak in the low‑q region (q = 0.02 Å^−^^1^). This suppression of the peak demonstrated that the incorporated lignin effectively inhibited nanoscale phase separation and local aggregation (Figure 2j). WAXS analysis was further used to evaluate the chain‐packing behavior and structural organization within the resin network. The characteristic scattering peak shifted toward a lower q value (Δq ≈ 0.012 Å^−^^1^), indicating an increase in the average interchain spacing after the incorporation of lignin. This structural variation suggested that lignin aromatic segments were successfully embedded into the polymer network, while the ester‐linked flexible units introduced stretchable buffer domains into the hydrogen‐bonding network. Integrating these multi‑scale observations, it was concluded that the resin synergistically formed an internal molecular network that was uniform, robust, and capable of efficient energy dissipation. This hierarchical structure established a critical foundation for the material's performance: as an adhesive, it enables high strength and reliable interfacial bonding, while as a composite matrix, it facilitates effective stress transfer and enhanced damage tolerance [34].

### 2.3 Application of Bio‐Based Epoxy Resins in Adhesives

**FIGURE 3:**
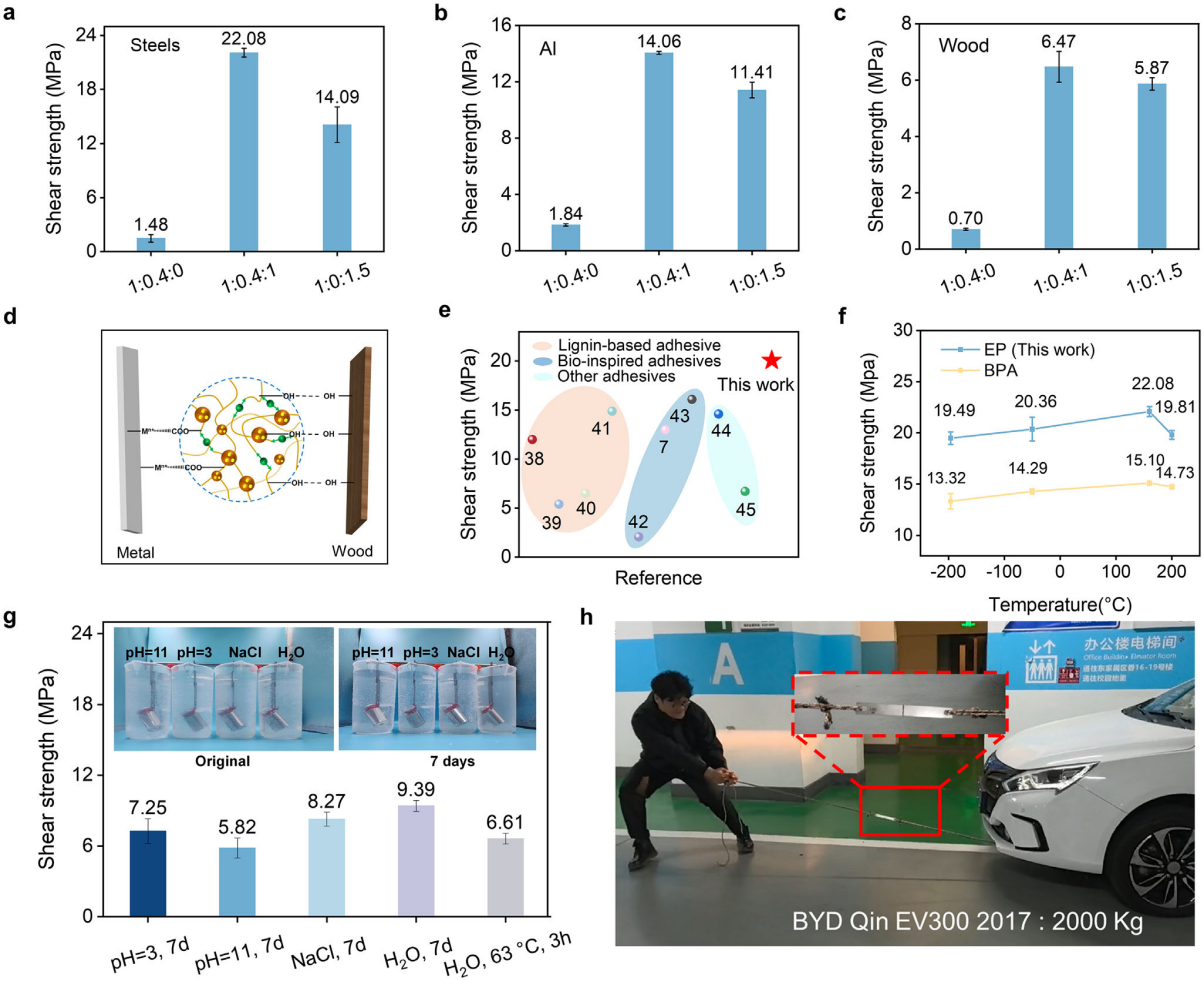
Application of bio‐based epoxy resins in adhesives. (a–c) The shear strength of adhesives on steel, Al, and wood substrates. (d) Schematic illustrations of the proposed adhesion mechanisms on metal and wood substrates. (e) Comparison of shear strengths between the bio‑based epoxy resin adhesives developed in this work and previously reported adhesives. (f) Shear performance of bonded steel joints after exposure to extreme conditions for 1 h. (g) Shear strength of the bio‑based epoxy adhesive for bonding steel (bonding area: 20 × 12 mm^2^; sustained load: 500 g) after one‑week immersion in various solvents. (h) Demonstration of the adhesive strength: a bonded joint dragging a 2000 kg car.

The bio‐based epoxy adhesive system achieved exceptional initial tack through a rationally designed dynamic cross‐linking mechanism, enabling multi‐substrate adhesion across diverse materials including glass, wood, plastic, rubber, metals, and polytetrafluoroethylene (PTFE) (Figure S19). The core of this performance lay in a three‐dimensional network architecture (Figure S20) constructed through synergistic reactions between epoxidized soybean oil, malic acid, and esterified lignin. Epoxide ring‐opening with malic acid generated ester‐bonded hydroxyl‐rich backbones, while lignin's aromatic rigidity enhanced cross‐linking density by restricting chain mobility. Crucially, esterified lignin simultaneously suppressed interfacial defects through covalent grafting and hydrogen bonding, with malic acid serving dual roles as both proton donor and cross‐linker [7]. Substrate‐specific performance analysis revealed striking differences: metal substrates (steel: 22.08 MPa; Al: 14.06 MPa) outperformed wood (6.47 MPa) (Figure 3a–c). This divergence originated from fundamental interfacial chemistry disparities (Figure 3d) [35]. Metal surfaces leveraged native oxide layers (Al_2_O_3_, Cr_2_O_3_) to form coordination complexes (50–400 kJ/mol) with resin carboxyl/epoxy groups, while their nonporous morphology ensured optimal adhesive wetting and continuous interfacial films [36]. Conversely, wood's hygroscopic fibrous structure promoted resin infiltration into cellulose micropores, creating mechanically inferior interphases that relied on low‐energy hydrogen bonds (2–30 kJ/mol) and amplified stress concentration under shear [37]. Compared to previously reported adhesives, the material developed in this work exhibited superior shear strength (Figure 3e) [7, 38, 39, 40, 41, 42, 43, 44, 45]. The adhesive maintained high bond strength even in extreme environments (−196 to 200 °C) and performed superiorly to the BPA (Figure 3f). Besides, short‐term solvent‐resistance tests under sustained loading showed that the resin possessed good chemical tolerance, as bonded stainless‐steel plates bearing a 500 g weight at one end maintained high shear strengths after one‐week immersion in various media, including alkaline solution (pH 11), acidic solution (pH 3), NaCl solution, and water (Figure 3g). This work establishes a sustainable high‐performance adhesive paradigm, combining universal bonding capability with metal‐specific superlative strength.

The developed bio‐based epoxy adhesive system demonstrated remarkable multifunctionality, combining robust environmental resistance with intelligent degradable properties. The adhesive exhibited extraordinary mechanical resilience, sustaining a 2.5 kg static load across a suspended span, dynamically lifting 120 kg adult (Figure S21), and successfully towing a 2000 kg vehicle (Figure 3h and Videos S3). Such capabilities highlight its engineered interfacial integrity and fatigue resistance, positioning it as a viable alternative for structural engineering and heavy‐load industrial applications. In daily life applications, traditional adhesives, while capable of securely fixing components, often encountered challenges such as difficult interfacial separation and adhesive residue contamination during item removal or repositioning. The bio‐based epoxy resin adhesive addressed critical limitations of conventional adhesives through pH‐responsive debonding functionality. The ESO–lignin–malic acid ternary bio‐based adhesive exhibited excellent initial adhesion to stainless‐steel substrates. After alkaline degradation and recycling, the lap‐shear strength gradually decreased with increasing recycling cycles, mainly due to ester‐bond hydrolysis‐induced network disruption, progressive chain fragmentation, and weakened interfacial interactions (Figure S22). To further illustrate the application relevance of alkali‐triggered debonding, a model demonstration was performed by bonding a stainless‐steel sheet bearing a school emblem onto a glass substrate (Figure S23). Targeted treatment with 1 M NaOH at 80°C for 30 min enabled residue‐free detachment through selective ester bond cleavage, while preserving the integrity of the glass substrate and allowing subsequent recoating with recovered adhesion performance. This environmentally responsive bonding/debonding mechanism significantly reduced maintenance costs, offering sustainable solutions for smart home systems, reusable electronics, and circular packaging design. By harmonizing extreme‐environment durability with eco‐conscious degradability, the adhesive bridges the gap between high‐performance industrial requirements and sustainable material innovation.

### 2.4 Applications in Epoxy Resin/Glass Fabrics Composites

**FIGURE 4:**
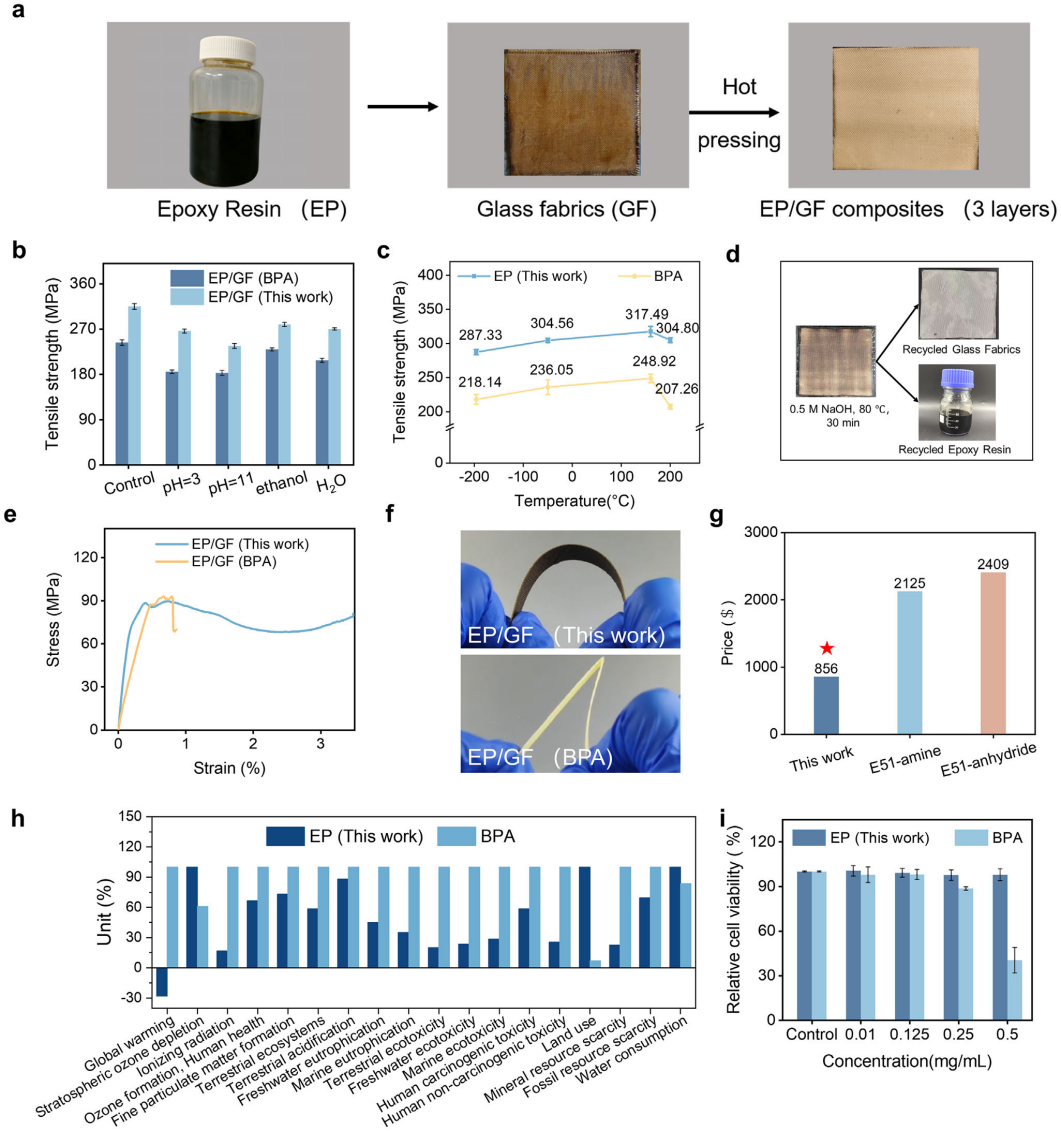
Synthesis and recyclability of Epoxy resin /glass fabric (EP/GF) composites. (a) Schematic representation of the preparation of EP/GF composites. (b) Results of tensile properties testing of EP/GF composites prepared from commercial BPA and bio‐based epoxy resins of the present study, and comparison of tensile properties after immersion for 24 h in pH 3, pH 11, H_2_O, and ethanol at room temperature. c, The tensile properties testing of EP/GF composites under extreme conditions for 1 h. (d) Digital images of EP/GF composites before and after decomposition of sodium hydroxide at 80°C. (e,f) Flexural strength and flexural durability of the EP/GF composites. (g) Market price comparison of bio‐based epoxy resin with commercial epoxy resin adhesives. (h) Environmental impact comparison of bio‐based epoxy resin and BPA epoxy resins per ton for each environmental impact. (i) Cytotoxicity.

Epoxy resin is an indispensable material for wind turbine blades. Its outstanding mechanical properties, fatigue resistance, and process adaptability are crucial for enhancing power generation efficiency, extending service life, and enabling industrial upgrading [46]. To evaluate the potential of bio‐based alternatives, we fabricated bio‐based EP/GF and compared them with commercial BPA‐based composites (Figure 4a and Figures S24 and S25). The bio‐based epoxy composites exhibited exceptional initial tensile strength (314.57 MPa) and environmental tolerance (Figure 4b), it maintained higher shear strength after 24‐h immersion in aggressive environments, including acid (pH 3), alkali (pH 11), ethanol, and water (Figure S26). Moreover, under extreme temperature conditions, the bio‐based EP/GF composite developed maintained high mechanical strength, and its stability was significantly superior to that of the commercial BPA epoxy resin system (Figure 4c). Critically, sodium hydroxide treatment facilitated selective decomposition and complete removal of the bio‐based resin matrix, achieving the structurally intact recovery of the glass fabric (Figure 4d). However, the composite remained stable under alkaline conditions at pH<11, demonstrating its selective degradability only under stronger bases (Figure S27). In addition, the flexural strength of the bio‐based EP/GF composite was comparable to that of the BPA‐based composite (approximately 90 MPa), indicating that its conventional mechanical properties reached a level equivalent to that of traditional BPA‐based materials. However, it is noteworthy that the bio‐based EP/GF composite also exhibited significant flexible strain capability, with the flexural strain increasing by approximately 250% (Figure 4e,f and Videos S4 and S5), a feature rarely observed in typically rigid fiber‐reinforced epoxy composites, demonstrating its unique structural character with a combination of rigidity and flexibility (Videos S4 and S5) [47]. Collectively, this bio‐based epoxy resin successfully addresses the critical recycling challenge of waste composites while ensuring the core performance required for wind turbine blades. It provides the wind power industry with a high‐performance, circular material option for environmentally sustainable development.

Economic viability served as a critical prerequisite for the large‐scale adoption of sustainable materials. Market data indicated that the price of domestically produced BPA epoxy resin was approximately $2,125 per ton for the amine‐cured type and as high as $2,409 per ton for the anhydride‐cured type. In contrast, the cost for the bio‐based epoxy adhesive developed in this study amounted to only $856 per ton (Figure 4g and Table S5). Notably, raw materials inputs dominated the cost structure, constituting over 90% of the total production expenses (Figure S28). Petroleum‐based feedstock prices exhibited significant volatility due to fluctuations in global energy markets, whereas bio‐based precursors demonstrated enhanced price stability through established agricultural supply chains. A cradle‐to‐gate life cycle assessment (LCA) was conducted to characterize the environmental impacts across production stages of bio‐based epoxy resin (Figures S29–S31 and Table S6). The carbon sequestration capacity of biomass feedstocks significantly reduced the global warming potential (GWP), while intensive agricultural practices and chemical processing remained critical bottlenecks due to their resource consumption and pollutant emissions. Besides, comparative analysis of environmental impacts between bio‐based and BPA epoxy resins (Figure 4h and Table S7) demonstrated that the bio‐based epoxy exhibited superior performance in GWP reduction and renewable resource utilization. However, it showed notable noncarbon environmental pressures from intensive agricultural production (fertilizer/water inputs), particularly in land use and water consumption. In contrast, the BPA‐based resin displayed more significant impacts in conventional environmental issues, including climate change, ecotoxicity, resource depletion, and human health risks (endocrine disruption), primarily due to its petroleum‐based feedstock dependency and BPA toxicity. Future improvements should focus on implementing precision agriculture (e.g., nitrogen/phosphorus optimization) and establishing circular resource systems to synergistically enhance the overall environmental performance of bio‐based resins and accelerate the replacement of BPA‐containing materials. In addition, the resin exhibited favorable biocompatibility, showing a cytotoxicity grade of 0–1 and a relative proliferation rate exceeding 90 % (Figure 4i), which benefited from the complete avoidance of toxic bisphenol A and the use of inherently safe bio‑based precursors. This cost advantage, when combined with the inherent carbon reduction benefits of bio‐based materials, is expected to accelerate the green transition of conventional adhesive industries.

## 3 Conclusions

In summary, a bio‐based high‐performance epoxy thermoset was constructed through a rigid–flexible synergistic design by integrating ESO, lignin, and malic acid. In this architecture, ESO imparted deformability, lignin provided rigid aromatic reinforcement, and maleic anhydride acted as a multifunctional bridge to improve compatibility and promote effective network formation. The resulting material exhibited a compelling combination of mechanical strength, ductility, and environmental adaptability, with strong performance in both structural adhesion and glass‐fiber‐reinforced composites. It also maintained stability under chemically aggressive environments and extreme temperatures, while displaying good biocompatibility and alkaline degradability. Life cycle and techno‐economic analyses further supported its reduced environmental impact and promising economic viability. This work provides a practical strategy for the development of high‐performance bio‐based epoxy thermosets and highlights their potential as sustainable alternatives for demanding structural applications. Nevertheless, long‐term ageing and accelerated ageing experiments should be further conducted in future work to systematically evaluate the long‐term service durability, degradation behavior, and failure mechanisms of this adhesive under prolonged and complex environmental conditions.

## 4 Experimental Section

### 4.1 Materials and Chemicals

Industrial lignin was supplied by Shandong Sun Holding Group Co., Ltd. (China). Epoxidized soybean oil(Product No. E107074‐500 mL, epoxy value of 6.0%–6.8%), malic acid (99%), maleic anhydride (99%), sodium hydroxide (98%), diethylenetriamine (99%) were procured from Shanghai Aladdin Biochemical Technology Co., Ltd. Hydrochloric acid (36%∼38%), ethanol (99.8%) and acetone (99.5%) were purchased from Modern Oriental (Beijing) Chemical Technology Co., Ltd. While N,N‐dimethylbenzylamine (99%) and 2‐ethyl‐4‐methylimidazole (96%) were obtained from Shanghai Macklin Biochemical Technology Co., Ltd. BPA epoxy resin E‐51 (epoxy value of 0.51) were purchased from Chuzhou Shenghui Electronic Materials Co., Ltd. All reagents were of analytical grade and used as received without further purification to ensure experimental reproducibility.

### 4.2 Synthesis of Esterified Lignin

The esterification reaction was conducted in a three‐necked round‐bottom flask equipped with a magnetic stirrer and reflux condenser. Initially, 5.0 g of industrial lignin was dispersed in acetone (mass ratio of lignin to acetone = 1:5, w/w) under constant stirring until complete dissolution. Subsequently, 3.0 g of maleic anhydride and 1.0 wt.% N,N‐dimethylbenzylamine (relative to the total mass of lignin and maleic anhydride) were sequentially introduced into the homogeneous reaction mixture. The system was heated to 80°C under vigorous mechanical stirring (500 rpm) for 8 h. The crude product was directly utilized for subsequent resin synthesis without purification. For structural characterization, a portion of the reaction mixture was cooled to ambient temperature, precipitated by dropwise addition into deionized water, and centrifuged (8000 rpm, 3 min) to isolate the modified lignin. The collected solid was subjected to freeze‐drying to remove residual solvents.

### 4.3 Preparation of Epoxy Resin Cross‐Linked Networks

Epoxidized soybean oil, malic acid, and esterified lignin were blended at mass ratios detailed in Table S1. The 1 wt.% 2‐ethyl‐4‐methylimidazole was incorporated as a curing accelerator, followed by mechanical stirring at 80°C and 500 rpm for 1 h to achieve homogeneous mixing. The pre‐polymerized resin was transferred to polytetrafluoroethylene (PTFE) molds (10 × 30 × 2 mm^3^) and subjected to vacuum degassing at 60°C for 2 h to eliminate solvents and microbubbles, the resin was cured at 120°C for 3 h and finally at 160°C for 3 h.

### 4.4 Adhesive Strength Analysis

Shear strength specimens were prepared using substrates of aluminum, stainless‐steel, and beech wood (80 mm × 20 mm × 2 mm) with an overlap area of 20 mm × 12 mm (240 mm^2^). Aluminum and stainless‐steel substrates were cleaned with 75% ethanol before bonding, whereas beech wood substrates were used as received without further surface pretreatment. Approximately 50.0 ± 0.5 mg of epoxy resin adhesive was applied to each joint and clamped with dovetail clamps to maintain a uniform adhesive thickness of 0.2 mm. The assemblies were vacuum cured at 160°C for 6 h to ensure complete solvent removal and adhesive cure. Environmental durability was evaluated using bonded stainless‐steel specimens after exposure to −196 to −200°C for 1 h or after immersion in pH 3 solution, pH 11 solution, ethanol, or water for one week under a 500 g load. The actual overlap dimensions were measured after curing using a calibrated ruler and used for strength calculation. Tensile lap‐shear tests were performed on a universal testing machine (UTM6503, Shenzhen Suns Technology Co., Ltd., China) equipped with a 5 kN load cell according to GB/T 7124‐2008, with a crosshead speed of 20 mm/min. Five replicates per substrate group were tested, and shear strength values were reported as mean ± standard deviation.

### 4.5 Preparation of EP/GF Composites

The epoxy resin was uniformly coated onto 100 mm × 100 mm glass fabrics using a roller coating method, followed by stacking three resin‐coated fabric layers and curing in a vulcanizing machine through a heating program (140°C/1 h, 160°C/2 h, 180°C/1 h) at a constant pressure of 2 MPa. For comparative analysis, EP/GF composites were simultaneously prepared using conventional BPA E‐51 under identical processing conditions. Tensile properties were evaluated using a universal testing machine (UTM6503) equipped with a 5 kN load cell, with lap shear strength measurements conducted at a crosshead speed of 5 mm/min. Specimens with dimensions of 100 mm × 10 mm × 0.5 mm were tested, and all reported data represent the average values from five replicate measurements.

## Author Contributions

J.Q. and J.W. conceived the project and designed experiments. J.Q. conducted all the experiments and testing analysis with assistance from X.H. X.Z analyzed the SAXS data. The manuscript was written and revised by J.Q at different stages. J.W. and T.Y. supervised the research project, reviewed and revised the manuscript at different stages, and are also responsible for funding acquisition, project administration, and ensuring data integrity. All authors discussed the results and commented on the manuscript.

## Conflicts of Interest

The authors declare no conflicts of interest.

## Supporting information




**Supporting File 1**: advs76659‐sup‐0001‐SuppMat.pdf.

**Supporting File 2**: advs76659‐sup‐0002‐SuppMat.zip.

## Data Availability

The data that support the findings of this study are available within the paper and Supplementary Information. Additional supporting data generated during the present study are available from the corresponding author on reasonable request.

## References

[1] Y. Zhang , X. Liu , M. Wan , Y. Zhu , and K. Zhang , “Recent Development of Functional Bio‐Based Epoxy Resins,” Molecules 29, no. 18 (2024): 4428, 10.3390/molecules29184428. 39339423 PMC11433883

[2] Y. Yuan , W. Lin , Y. Xiao , B. Yu , and W. Wang , “Flame‐retardant Epoxy Thermosets Derived from Renewable Resources: Research Development and Future Perspectives,” Journal of Materials Science & Technology 195 (2024): 29–40, 10.1016/j.jmst.2024.02.006.

[3] C. Gao , H. He , W. Qiu , et al., “Oxidative Stress, Endocrine Disturbance, and Immune Interference in Humans Showed Relationships to Serum Bisphenol Concentrations in a Dense Industrial Area,” Environmental Science & Technology 55, no. 3 (2021): 1953–1963, 10.1021/acs.est.0c07587. 33496180

[4] E. Buoso , M. Masi , R. V. Limosani , et al., “Endocrine Disrupting Toxicity of Bisphenol A and Its Analogs: Implications in the Neuro‐immune Milieu,” Journal of Xenobiotics 15, no. 1 (2025): 13, 10.3390/jox15010013. 39846545 PMC11755641

[5] X. Fang , N. Tian , W. Hu , et al., “Dynamically Cross‐Linking Soybean Oil and Low‐Molecular‐Weight Polylactic Acid toward Mechanically Robust, Degradable, and Recyclable Supramolecular Plastics,” Advanced Functional Materials 32, no. 46 (2022): 2208623, 10.1002/adfm.202208623.

[6] B. Zhang , P. Zhang , G. Zhang , C. Ma , and G. Zhang , “Sterically Hindered Oleogel‐Based Underwater Adhesive Enabled by Mesh‐Tailoring Strategy,” Advanced Materials 36, no. 29 (2024): 2313495, 10.1002/adma.202313495. 38683961

[7] C. R. Westerman , B. C. McGill , and J. J. Wilker , “Sustainably Sourced Components to Generate High‐Strength Adhesives,” Nature 621, no. 7978 (2023): 306–311, 10.1038/s41586-023-06335-7. 37704765

[8] J. Pansumdaeng , S. Kuntharin , V. Harnchana , and N. Supanchaiyamat , “Fully Bio‐based Epoxidized Soybean Oil Thermosets for High Performance Triboelectric Nanogenerators,” Green Chemistry 22, no. 20 (2020): 6912–6921, 10.1039/d0gc01738h.

[9] J. Sternberg , O. Sequerth , and S. Pilla , “Green Chemistry Design in Polymers Derived from Lignin: Review and Perspective,” Progress in Polymer Science 113 (2021): 101344, 10.1016/j.progpolymsci.2020.101344.

[10] L. Yan , A. J. Huertas‐Alonso , H. Liu , L. Dai , C. Si , and M. H. Sipponen , “Lignin Polymerization: Towards High‐Performance Materials,” Chemical Society Reviews 54, no. 14 (2025): 6634–6651, 10.1039/d4cs01044b. 40491312 PMC12150016

[11] S. Zhou , K. Huang , X. Xu , et al., “Rigid‐and‐Flexible, Degradable, Fully Biobased Thermosets from Lignin and Soybean Oil: Synthesis and Properties,” ACS Sustainable Chemistry & Engineering 11, no. 8 (2023): 3466–3473, 10.1021/acssuschemeng.2c06990.

[12] P. S. Sejati , F. Obounou Akong , F. Fradet , and P. Gérardin , “Understanding the Thermoplasticization Mechanism of Wood via Esterification with Fatty Acids: A Comparative Study of the Reactivity of Cellulose, Hemicelluloses and Lignin,” Carbohydrate Polymers 324 (2024): 121542, 10.1016/j.carbpol.2023.121542. 37985114

[13] R. Tian , C. Wang , W. Jiang , et al., “Biodegradable, Strong, and Hydrophobic Regenerated Cellulose Films Enriched with Esterified Lignin Nanoparticles,” Small 20, no. 33 (2024): 2309651, 10.1002/smll.202309651. 38530065

[14] T. Li , Y. Yin , S. Wu , H. Ma , and F. Zhang , “Effect of Pre‐Acetylation of Hydroxyl Functional Groups by Choline Chloride/Acetic Anhydride on Subsequent Lignin Pyrolysis,” Bioresource Technology 317 (2020): 124034, 10.1016/j.biortech.2020.124034. 32829115

[15] L. Szabó , R. Milotskyi , H. Ueda , T. Tsukegi , N. Wada , and K. Takahashi , “Controlled Acetylation of Kraft Lignin for Tailoring Polyacrylonitrile‐Kraft Lignin Interactions Towards the Production of Quality Carbon Nanofibers,” Chemical Engineering Journal 405 (2021): 126640, 10.1016/j.cej.2020.126640.

[16] L.‐Y. Liu , Q. Hua , and S. Renneckar , “A Simple Route to Synthesize Esterified Lignin Derivatives,” Green Chemistry 21, no. 13 (2019): 3682–3692, 10.1039/c9gc00844f.

[17] D. Zhang , K. Jin , K. H. Lim , S. Jie , W.‐J. Wang , and X. Yang , “Eco‐Friendly Cellulose Nanofibrils With High Surface Charge and Aspect Ratio for Nanopaper Films With Ultrahigh Toughness and Folding Endurance,” Green Chemistry 25, no. 12 (2023): 4696–4704, 10.1039/d3gc00632h.

[18] H. Yang , Y. Yang , L. Deng , et al., “Lignin‐Tannic Acid Epoxy Resins Based on Dynamic Covalent Bonding of β‐hydroxy Esters with Closed‐Loop Recyclability, Exceptional Adhesive Properties and Excellent Photothermal Capacity,” International Journal of Biological Macromolecules 321 (2025): 146084, 10.1016/j.ijbiomac.2025.146084. 40695433

[19] Z. Gong , G. Yang , L. Huang , L. Chen , X. Luo , and L. Shuai , “Phenol‐Assisted Depolymerisation of Condensed Lignins to Mono‐/Poly‐Phenols and Bisphenols,” Chemical Engineering Journal 455 (2023): 140628, 10.1016/j.cej.2022.140628.

[20] E. Subbotina , P. Olsén , M. Lawoko , and L. A. Berglund , “Maleated Technical Lignin Thermosets and Biocomposites Designed for Degradation,” ACS Sustainable Chemistry & Engineering 12, no. 9 (2024): 3632–3642, 10.1021/acssuschemeng.3c06741.

[21] Y. Fan , C. Liu , X. Kong , Y. Han , M. Lei , and R. Xiao , “A New Perspective on Polyethylene‐Promoted Lignin Pyrolysis with Mass Transfer and Radical Explanation,” Green Energy & Environment 7, no. 6 (2022): 1318–1326, 10.1016/j.gee.2021.02.004.

[22] B. M. Upton and A. M. Kasko , “Strategies for the Conversion of Lignin to High‐Value Polymeric Materials: Review and Perspective,” Chemical Reviews 116, no. 4 (2016): 2275–2306, 10.1021/acs.chemrev.5b00345. 26654678

[23] X. Yue , J. Lin , O. Mankinen , et al., “Lignin Dissolution and Direct Ultrasmall‐Lignin‐Nanoparticle Formation in Acidic and Alkaline Deep Eutectic Solvents: A Molecular‐Level Insight,” Angewandte Chemie International Edition 64, no. 30 (2025): 202505975, 10.1002/anie.202505975. PMC12281085 40400210

[24] Y. Li , M. Zhou , Y. Pang , and X. Qiu , “Lignin‐Based Microsphere: Preparation and Performance on Encapsulating the Pesticide Avermectin,” ACS Sustainable Chemistry & Engineering 5, no. 4 (2017): 3321–3328, 10.1021/acssuschemeng.6b03180.

[25] M. Kim , Y. A. Lee , J. Wu , et al., “Fabrication of Hydrophobic Lignin‐Based Films Through Tandem Chemical Modification and Plasma Treatment,” ACS Applied Polymer Materials 7, no. 1 (2024): 503–511, 10.1021/acsapm.4c03574.

[26] J. Sun , H. Huang , W. Wang , et al., “Ultratough, Processable Bioplastics Enabled by Triple Interlocking of Lignin and Cellulose,” ACS Nano 19, no. 32 (2025): 29360–29371, 10.1021/acsnano.5c06221. 40785524

[27] C. Cai , K. Hirth , R. Gleisner , H. Lou , X. Qiu , and J. Y. Zhu , “Maleic Acid as a Dicarboxylic Acid Hydrotrope for Sustainable Fractionation of Wood at Atmospheric Pressure and ≤100°C: Mode and Utility of Lignin Esterification,” Green Chemistry 22, no. 5 (2020): 1605–1617, 10.1039/c9gc04267a.

[28] E. Bellinetto , N. Fumagalli , M. Astorri , S. Turri , and G. Griffini , “Elucidating the Role of Lignin Type and Functionality in the Development of High‐Performance Biobased Phenolic Thermoset Resins,” ACS Applied Polymer Materials 6, no. 2 (2024): 1191–1203, 10.1021/acsapm.3c02136. 38299121 PMC10825815

[29] H. Liu , X. Liu , L. Wang , et al., “Organic‐Inorganic Hybrid Epoxy Vitrimers With Excellent Thermal‐Mechanical Stability Based on Carboxylic‐Acid Type Carbon Dots as Curing Agent: Flame Retardancy and Reprocessing,” Composites Part B: Engineering 297 (2025): 112316, 10.1016/j.compositesb.2025.112316.

[30] H. Xie , F. Bao , H. Zhang , et al., “Ultratough, Robustness, and Reprocessable Thermoset Epoxy Resins Synergistically Enhanced by Hierarchical Coordination Interactions and Dynamic Covalent Networks,” Advanced Functional Materials 34, no. 48 (2024): 2408411, 10.1002/adfm.202408411.

[31] C. Yang , T. Shen , Z. Tan , et al., “Lignin Modification for in‐Situ Cured Lignin‐Maleyl Network in Semi‐Crystalline Polyamide/Lignin Shape Memory Composites,” Industrial Crops and Products 197 (2023): 116665, 10.1016/j.indcrop.2023.116665.

[32] S. Cai , X. Zhang , Z. Wang , and H. Xia , “Carbon Fiber‐Reinforced Dynamically Cross‐Linked Epoxy Resin Composites With Excellent Self‐Healing and Recycling Performance via Autocatalyzed β‐Hydroxyl Ester Bonds,” Industrial & Engineering Chemistry Research 64, no. 1 (2024): 369–381, 10.1021/acs.iecr.4c02685.

[33] W. Wu , H. Feng , L. Xie , et al., “Reprocessable and Ultratough Epoxy Thermosetting Plastic,” Nature Sustainability 7, no. 6 (2024): 804–811, 10.1038/s41893-024-01331-9.

[34] Y. Zhang , J. Li , X. Wu , et al., “Simultaneously Reinforcing and Toughening of Shape‐Memory Epoxy Resin With Carboxylated Lignosulfonate: Facile Preparation and Effect Mechanism,” International Journal of Biological Macromolecules 217 (2022): 243–254, 10.1016/j.ijbiomac.2022.07.047. 35835301

[35] T. Miyata , Y. K. Sato , Y. Kawagoe , et al., “Effect of Inorganic Material Surface Chemistry on Structures and Fracture Behaviours of Epoxy Resin,” Nature Communications 15, no. 1 (2024): 1898, 10.1038/s41467-024-46138-6. PMC10923874 38459006

[36] C. Ochoa‐Putman and U. K. Vaidya , “Mechanisms of Interfacial Adhesion in Metal–Polymer Composites—Effect of Chemical Treatment,” Composites Part A: Applied Science and Manufacturing 42, no. 8 (2011): 906–915, 10.1016/j.compositesa.2011.03.019.

[37] S. Luo , Y. Sun , Y. Zhu , et al., “A New Strategy for the Preparation of Wood‐Epoxy Resin Composites Reinforced with Controllable Osmotic Interfaces,” Chemical Engineering Journal 484 (2024): 148880, 10.1016/j.cej.2024.148880.

[38] H. Wang , S. Tan , Z. Su , M. Li , X. Hao , and F. Peng , “Perforin‐Mimicking Molecular Drillings Enable Macroporous Hollow Lignin Spheres for Performance‐Configurable Materials,” Advanced Materials 36, no. 15 (2024): 2311073, 10.1002/adma.202311073. 38199249

[39] T. Zou , M. H. Sipponen , A. Henn , and M. Österberg , “Solvent‐Resistant Lignin‐Epoxy Hybrid Nanoparticles for Covalent Surface Modification and High‐Strength Particulate Adhesives,” ACS nano 15, no. 3 (2021): 4811–4823, 10.1021/acsnano.0c09500. 33593063 PMC8023795

[40] S. Zhang , T. Liu , C. Hao , et al., “Preparation of a Lignin‐Based Vitrimer Material and Its Potential Use for Recoverable Adhesives,” Green Chemistry 20, no. 13 (2018): 2995–3000, 10.1039/c8gc01299g.

[41] J. Liu , Y. Liu , Z. Hou , et al., “One‐Step Synthesis of Waterborne Epoxidized Lignin Nanoparticles With High Epoxy Value and Stability for High‐Strength Adhesives,” ACS Sustainable Chemistry & Engineering 12, no. 42 (2024): 15376–15386, 10.1021/acssuschemeng.4c02695.

[42] X. Huang , Y. Chen , X. Lin , et al., “A Reusable Soy Protein Adhesive With Enhanced Weather Resistance Through Construction of a Cutin‐Like Structure,” Cell Reports Physical Science 5, no. 6 (2024): 102024, 10.1016/j.xcrp.2024.102024.

[43] C. J. Higginson , K. G. Malollari , Y. Xu , A. V. Kelleghan , N. G. Ricapito , and P. B. Messersmith , “Bioinspired Design Provides High‐Strength Benzoxazine Structural Adhesives,” Angewandte Chemie International Edition 58, no. 35 (2019): 12271–12279, 10.1002/anie.201906008. 31276607 PMC6772131

[44] H. Rong , P. Zhao , M. Lu , et al., “On‐Demand Detachable and Reuseable Adhesives Assembled by Organic‐Silicon Nanogels with Wide Applicability and Excellent Harsh‐Environment Resistance,” Chemical Engineering Journal 498 (2024): 155566, 10.1016/j.cej.2024.155566.

[45] L.‐Y. Li , W. Chen , C.‐H. Hu , Y.‐D. Li , and J.‐B. Zeng , “One‐Pot Solvent‐Free Synthesis of Imine‐Based Epoxidized Soybean Oil Vitrimers for Sustainable Adhesives,” ACS Sustainable Chemistry & Engineering 13, no. 1 (2024): 547–558, 10.1021/acssuschemeng.4c08530.

[46] A. Ahrens , A. Bonde , H. Sun , et al., “Catalytic Disconnection of C–O Bonds in Epoxy Resins and Composites,” Nature 617, no. 7962 (2023): 730–737, 10.1038/s41586-023-05944-6. 37100913 PMC10208972

[47] J. Zhang , C. Jiang , G. Deng , et al., “Closed‐Loop Recycling of Tough Epoxy Supramolecular Thermosets Constructed With Hyperbranched Topological Structure,” Nature Communications 15, no. 1 (2024): 4869, 10.1038/s41467-024-49272-3. PMC11161517 38849328

