# Supplementary material for: A Sustainable Bio‐Based Epoxy Thermoset as a High‐Performance Alternative to BPA‐Based Resins
Source: Adv Sci (Weinh). 2026 Jul 20;13(57):e76659. doi: 10.1002/advs.76659 (PMC13383703; doi:10.1002/advs.76659)
Supplement: Supplementary file 1 [file ADVS-13-e76659-s002.pdf]

Supporting Information

A Sustainable Bio-Based Epoxy Thermoset as a High-Performance Alternative to BPA-Based Resins

Jiyun Qi, Xinyi Hui, Xipeng Zhang, Jia-Long Wen, Tong-Qi Yuan**

J Qi, X Hui, X Zhang, J Wen, T Yuan

State Key Laboratory of Efficient Production of Forest Resources, Beijing Forestry University, Beijing 100083, China

Beijing Key Laboratory of Lignocellulosic Chemistry, Beijing Forestry University, Beijing 100083, China

E-mail: wenjialong@bjfu.edu.cn; ytq581234@bjfu.edu.cn

Characterizations

Chemical structure analysis

The chemical structure of the samples was analyzed using FTIR (Thermo Scientific Nicolet iN10, USA) equipped with a liquid nitrogen-cooled MCT detector, ranged from 4000 to 700 cm^{-1}. The molecular structure of lignin was analyzed using a Bruker Avance III HD 400 MHz NMR spectrometer (Bruker Scientific Instruments, USA). Lignin samples were prepared in DMSO-d_6 or derivatized for ^{31}P NMR, and all measurements were performed at 298 K.

Gel permeation chromatography (GPC)

The molecular weights (Mn and Mw) of lignin before and after modification were determined by GPC (Agilent 1200, US). 4 mg samples were dissolved in 2 mL THF, filtered through a 0.45 μm membrane, and injected into the system. THF was used as the mobile phase at 0.5 mL/min.

Solubility Test

The solubility of industrial lignin and its esterified derivative was evaluated in four organic solvents: methanol, ethanol, acetone, and tetrahydrofuran (THF). Specifically, 5.0 ± 0.1 mg of each sample was added to a glass vial containing 10.0 mL of solvent. The mixtures were magnetically stirred at 150 rpm and 25 ± 1 °C for 10 min to ensure complete dispersion. Solubility was visually assessed under ambient light conditions.

Thermogravimetric analysis (TGA)

The thermal stability of the samples was evaluated using thermogravimetric analysis (TGA) performed on a DTG-60 analyzer (Shimadzu Corporation, Japan). All

measurements were carried out under a nitrogen purge (50 mL min^{-1} flow rate) with a controlled heating ramp of 10 °C min^{-1} from 30 °C to 800 °C.

Differential scanning calorimeter analysis (DSC)

The curing behavior of the epoxy resin was investigated using differential scanning calorimetry (DSC 60, Shimadzu, Japan) under a nitrogen atmosphere with a constant purge flow rate of 20 mL/min. Approximately 5 mg samples were sealed in standard aluminum crucibles and subjected to dynamic heating scans from 25 to 180 °C at a heating rate of 5 °C/min.

Gel content test

The gel content of the cured epoxy resins was determined by solvent extraction in 1,4-dioxane. Approximately 80 mg of sample (m_0) was swollen in 4 mL of solvent at 100 °C for 12 h. The insoluble fraction was then collected by filtration, washed thoroughly with acetone, and dried under vacuum at 60 °C for 24 h to a constant mass (m_1). The gel content was calculated as $(m_1 / m_0) \times 100\%$.

Water Contact angle

Dynamic contact angles were measured using a contact angle goniometer (SL200KS, KINO Scientific Instruments, USA) with a high-speed camera and automated dispensing system. Deionized water (3 μL) was used as the probing liquid under ambient conditions. Contact angles were monitored for 5 minutes at 1 fps, with averages calculated from at least three independent measurements.

Breakdown strength.

The breakdown strength was determined using a precision dielectric breakdown tester

(BDJC-50KV, Beijing Beiguang Jingyi Instrument Co., Ltd., China). Tests were performed by applying AC and DC voltages at a ramp rate of 2 kV/s until failure occurred, employing a sphere-sphere electrode configuration with a diameter of 20 mm. It was calculated using the equation Eb = U / d, where U is the breakdown voltage and d is the sample thickness.

Cytotoxicity assay.

Bio-based epoxy resin and commercial BPA epoxy resin were used as test materials and pretreated by autoclaving (121 °C, 20 min). Subsequently, robustly growing L-929 cells in the logarithmic growth phase (cultured for 48 hours) were selected as the experimental cells. The cells were treated with material extract solutions at concentrations of 0.5, 0.25, 0.125, and 0.01 mg/mL, respectively, and incubated in a 5% CO_2 atmosphere at 37 °C for 48 hours. Cell proliferation rates were assessed at each observation time point using the CCK-8 assay.

Scanning Electron Microscopy (SEM)

The fracture surface morphology of various samples was characterized using a scanning electron microscope (SEM, S-3400N, Hitachi, Japan). Prior to SEM observation, the epoxy resin samples were cryogenically fractured in liquid nitrogen to obtain clean fracture surfaces. To enhance conductivity, the fractured samples were sputter-coated with a thin layer of gold (~10 nm) using a gold sputter coater.

Small-angle X-ray scattering (SAXS) and wide-angle X-ray scattering (WAXS).

The microstructure of the epoxy resins was characterized using small-angle and wide-angle X-ray scattering (SAXS/WAXS). Measurements were conducted on an Anton

Paar SAXSpoint 2.0 system (Anton Paar, Austria) equipped with a Cu Kα microfocus source (λ = 1.54189 Å) and a two-dimensional EIGER R detector. The scattering vector q was calibrated with a silver behenate standard. The obtained two-dimensional patterns were processed through standard azimuthal integration, background subtraction, and normalization to yield the one-dimensional intensity profiles I(q).

Life-cycle assessment (LCA).

LCA was conducted in accordance with ISO 14040/14044 standards, with 1 ton of bio-based epoxy resin being defined as the functional unit. The SimaPro 9.0 software platform was utilized to integrate data from both the Ecoinvent database and literature sources, while the ReCiPe 2016 midpoint (H) method was employed to evaluate 18 environmental impact categories.

Table S1. The reaction conditions for the epoxy resin cross-linked networks.

Samples	ESO (g)	malic acid (g)	esterified lignin (g)	Temperature (℃)	Time (h)
1:0.4:0	1	0.4	0	80	1
1:0.4:1	1	0.4	1	80	1
1:0:1.5	1	0	1.5	80	1

Table S2. Quantification of the DEL and EL by quantitative ^{31}P NMR（$mmol\ g^{-1}$）.

Samples	Aliphatic OH	Syringyl OH	Guaiacyl OH		p-Hydroxy phenyl OH	Carboxylic group
			C[a]	NC[b]		
DEL	1.21	2.57	0.46	0.67	0.13	0.23
EL	0.42	2.09	0.33	0.51	0.08	0.61

[a]C, condensed. [b]NC, non-condensed.

Table S3. Thermal characteristics of control and esterified lignin.

**Sample**	**$T_{5\%}$/°C**	**$T_{10\%}$/°C**	**$T_{50\%}$/°C**	**Carbon Residual/%**
Control sample	268.93	311.26	656.01	47.45
Esterified lignin	184.27	241.09	488.03	39.88

Table S4. Thermal characteristics of bio-based epoxy resins.

Sample	$T_{5\%}$/°C	$T_{10\%}$/°C	$T_{50\%}$/°C	Carbon Residual/%
1:0.4:0	283.14	332.96	405.10	3.79
1:0.4:1	217.27	243.08	394.55	16.70
1:0:1.5	221.47	254.01	395.76	14.57

Table S5. Detailed operating costs for producing 1ton bio-based epoxy resin.

Raw material	Quantity	Unit	Price
Step 1: Synthesis of esterified lignin			
Lignin	113	kg	4000￥/ton
Maleic anhydride	68	kg	4950￥/ton
Acetone	566	kg	4114￥/ton
Energy	400	kwh	0.4￥/kwh
N,N-dimethylbenzylamine	1.81	Kg	14500￥/ton
Cost: 452 + 336.6 + 2328.5 + 160 + 26.25 = 3303￥			
Step 2: Preparation of epoxy resin cross-linked networks			
Epoxidized soybean oil	181	kg	7600￥/ton
Malic acid	72	kg	10500￥/ton
Esterified lignin	Step 1	/	/
Energy	30	kwh	0.4￥/kwh
2-ethyl-4-methylimidazole	4.34	kg	150000￥/ton
Cost: 1375.6 + 756 + 3303 + 12 + 651= 6098￥			
Fixed operating cost：Estimated at 5% of the total cost of raw materials and electricity 305￥			
Total: 6098 +305=6403￥/ton or 856＄/ton			

Table S6. Environmental impact values for different scenarios.

Impact category	Unit	Total	Epoxidized soy oil	Esterified lignin	Malic acid	Electricity
Global warming	kg CO_2 eq	-1215.0817	-1414.3285	49.6068	118.7088	30.9312
Stratospheric ozone depletion	kg CFC11 eq	0.0017	0.0024	0.0000	-0.0007	0.0000
Ionizing radiation	kBq Co-60 eq	29.8572	25.9192	3.7326	0.1612	0.0442
Ozone formation, Human health	kg NOx eq	7.6432	3.7539	3.5605	0.2429	0.0860
Fine particulate matter formation	kg $PM_{2.5}$ eq	5.0849	3.0133	1.9507	0.0609	0.0601
Ozone formation, Terrestrial ecosystems	kg NOx eq	8.0006	3.8221	3.8491	0.2431	0.0862
Terrestrial acidification	kg SO_2 eq	12.5463	6.7140	5.6730	0.0130	0.1463
Freshwater eutrophication	kg P eq	0.6832	0.3072	0.3529	0.0174	0.0056
Marine eutrophication	kg N eq	0.0658	0.0578	0.0065	0.0012	0.0003
Terrestrial ecotoxicity	kg 1,4-DCB	2198.1165	1690.1351	448.7041	44.6063	14.6710
Freshwater ecotoxicity	kg 1,4-DCB	33.1271	26.2553	5.9492	0.6625	0.2601
Marine ecotoxicity	kg 1,4-DCB	45.0689	35.3498	8.3965	0.9560	0.3667
Human carcinogenic toxicity	kg 1,4-DCB	88.0235	56.1656	28.4497	2.5718	0.8364
Human non-carcinogenic toxicity	kg 1,4-DCB	854.2623	669.7255	162.3659	15.7735	6.3974
Land use	m^2a crop eq	831.4037	826.7847	2.9175	1.3378	0.3638
Mineral resource scarcity	kg Cu eq	2.3126	1.9720	0.3089	0.0242	0.0076
Fossil resource scarcity	kg oil eq	1398.2073	463.2766	930.3893	-1.4815	6.0230
Water consumption	m^3	62.4022	23.4190	37.9872	0.9224	0.0736

Table S7. Environmental impact comparison of bio-based epoxy resin and BPA epoxy resins per ton for each environmental impact.

Impact category	Unit	Bio-based epoxy resins	BPA
Global warming	kg CO_2 eq	-1215.0817	4332.1142
Stratospheric ozone depletion	kg CFC11 eq	0.0017	0.0010
Ionizing radiation	kBq Co-60 eq	29.8572	178.5358
Ozone formation, Human health	kg NOx eq	7.6432	11.5177
Fine particulate matter formation	kg $PM_{2.5}$ eq	5.0849	6.9512
Ozone formation, Terrestrial ecosystems	kg NOx eq	8.0006	13.6658
Terrestrial acidification	kg SO_2 eq	12.5463	14.2476
Freshwater eutrophication	kg P eq	0.6832	1.5153
Marine eutrophication	kg N eq	0.0658	0.1868
Terrestrial ecotoxicity	kg 1,4-DCB	2198.1165	11027.0764
Freshwater ecotoxicity	kg 1,4-DCB	33.1271	140.9251
Marine ecotoxicity	kg 1,4-DCB	45.0689	158.5182
Human carcinogenic toxicity	kg 1,4-DCB	88.0235	150.6490
Human non-carcinogenic toxicity	kg 1,4-DCB	854.2623	3346.6504
Land use	m^2a crop eq	831.4037	59.0296
Mineral resource scarcity	kg Cu eq	2.3126	10.1657
Fossil resource scarcity	kg oil eq	1398.2073	2015.1246
Water consumption	m^3	62.4022	52.0279

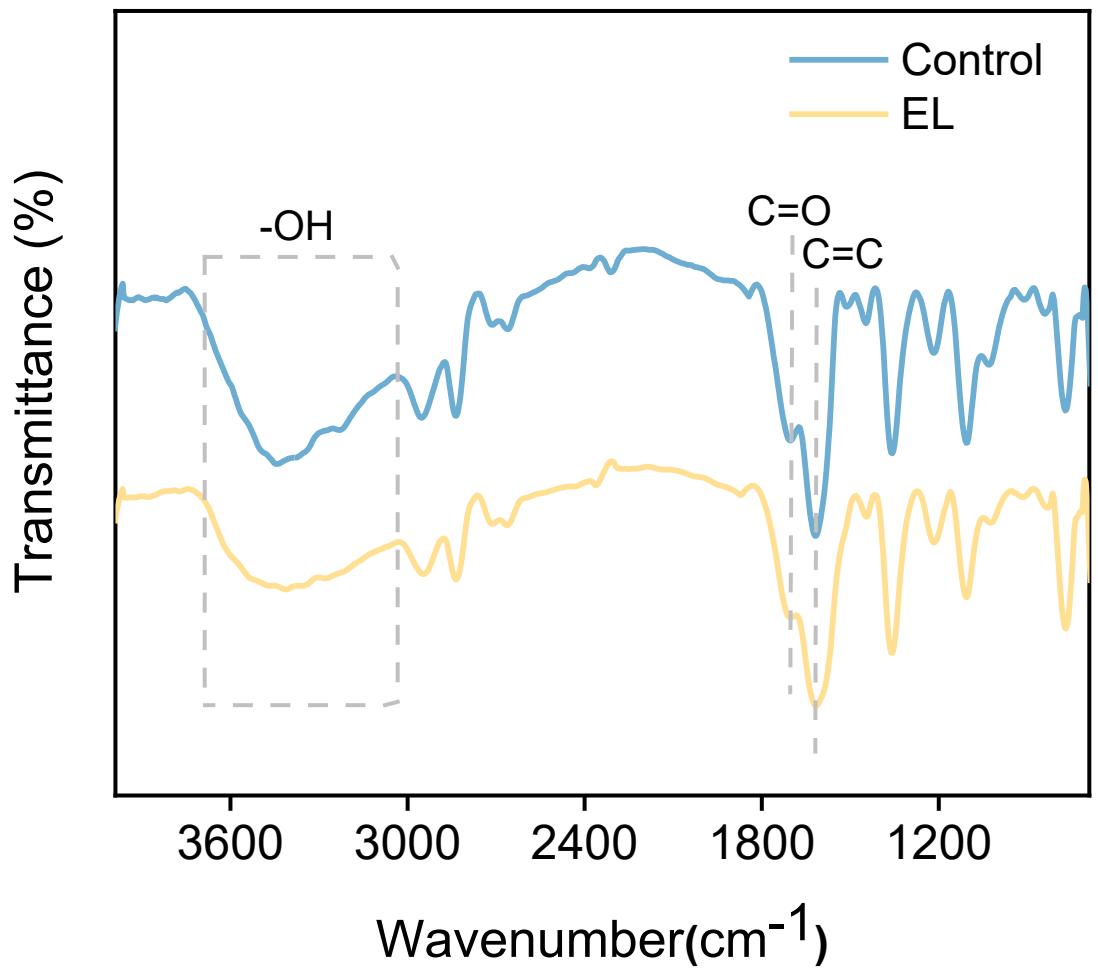

Figure S1. FTIR spectra of control lignin and maleic anhydride-modified esterified lignin. Selective esterification between lignin and maleic anhydride was confirmed by the decreased -OH peak intensity (3000–3600 cm^{-1}) and the enhanced C=O stretching peak of ester groups (~1730 cm^{-1}). The retention of aromatic C=C vibration peak revealed the intact aromatic backbone of lignin after modification, and the obtained EL exhibited enhanced chemical reactivity and structural integrity.

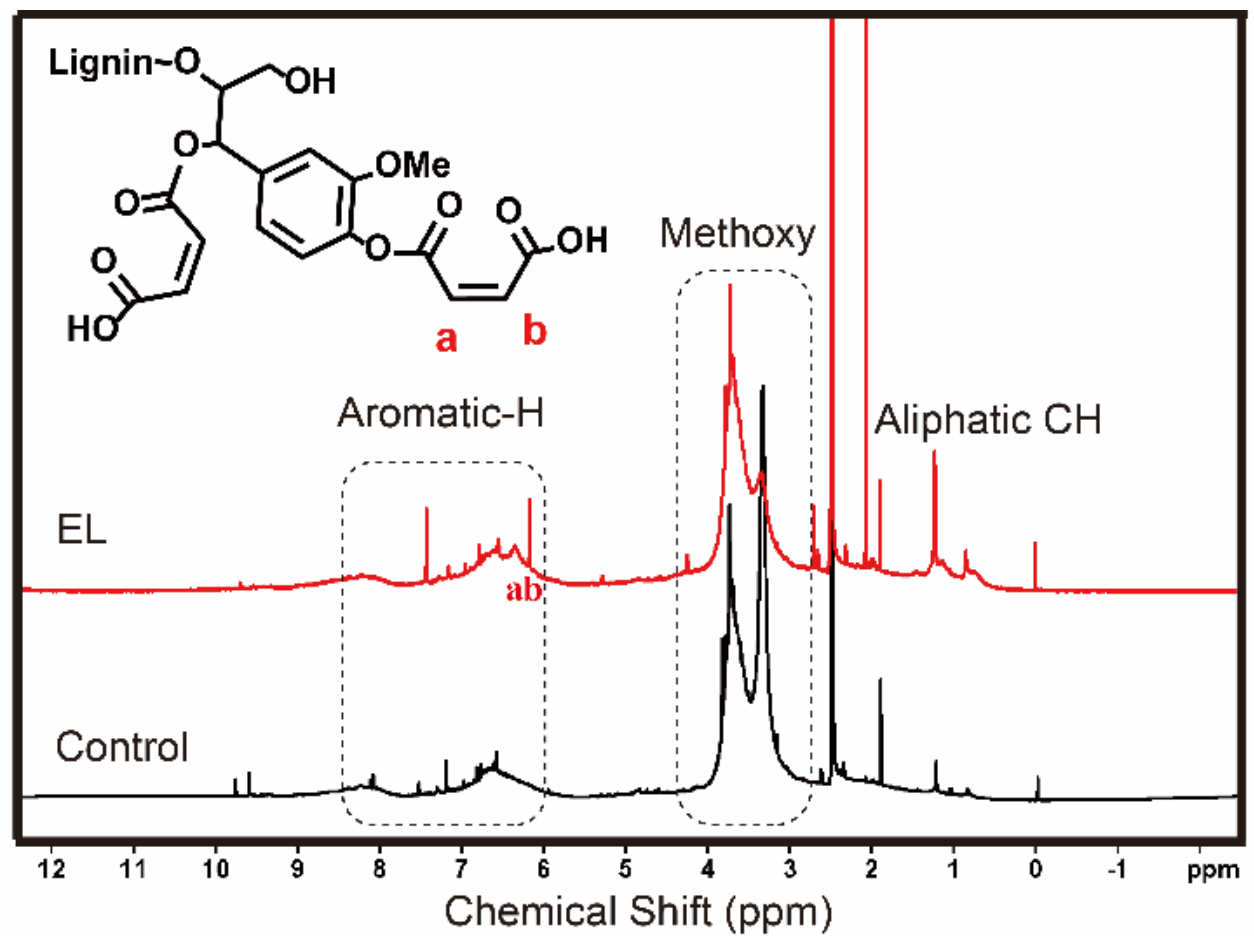

Figure S2. ^{1}H NMR spectra of control lignin and maleic anhydride-esterified lignin. New proton peaks assigned to the olefinic hydrogen of grafted maleic anhydride appeared in the aromatic hydrogen region (6.0–8.0 ppm), and the increased signal intensity of aliphatic protons (0–3.0 ppm) further verified the successful grafting of maleic anhydride onto lignin via selective esterification.

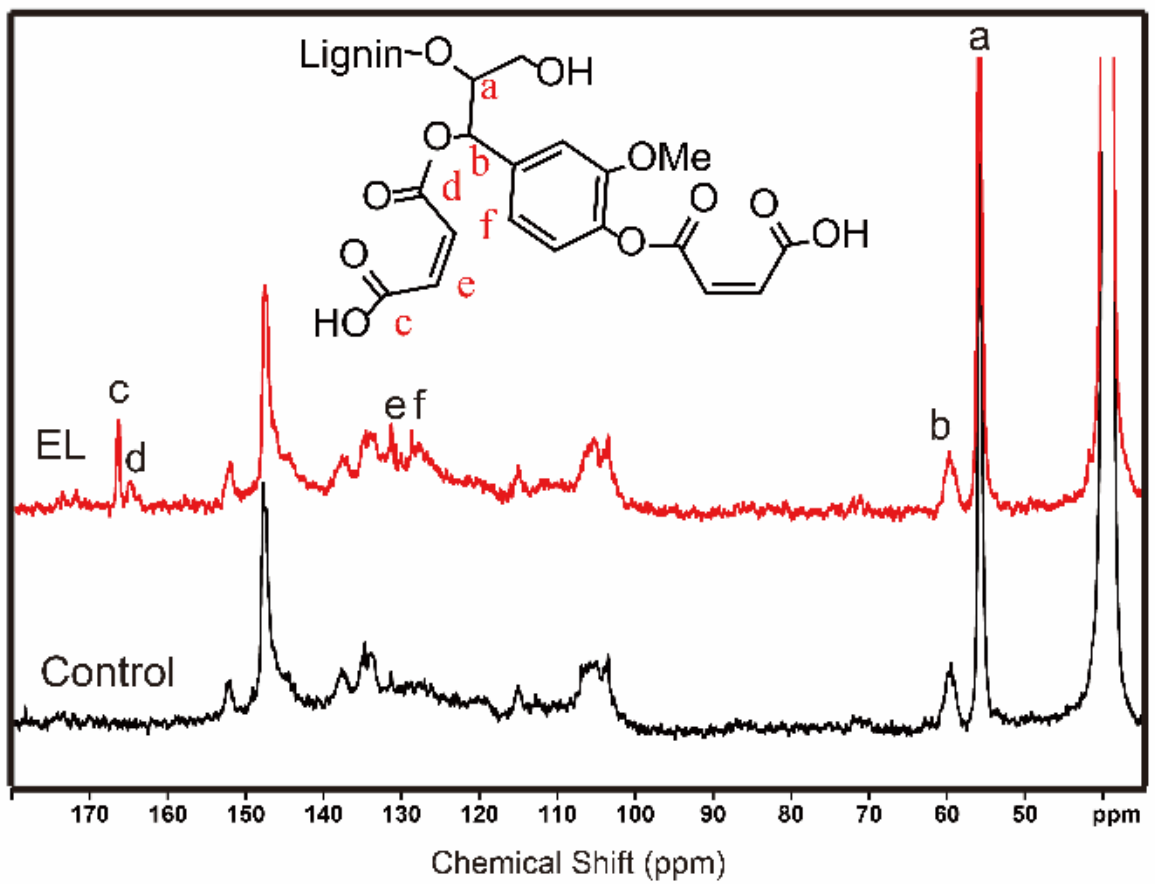

Figure S3. ^{13}C NMR of control and esterified lignin. The newly appeared ester and carboxyl carbon peaks at 160–175 ppm verified the successful grafting of maleic anhydride onto lignin via esterification. The retention of lignin's aromatic and aliphatic carbon signals confirmed that the intrinsic skeleton of lignin remained intact after selective modification.

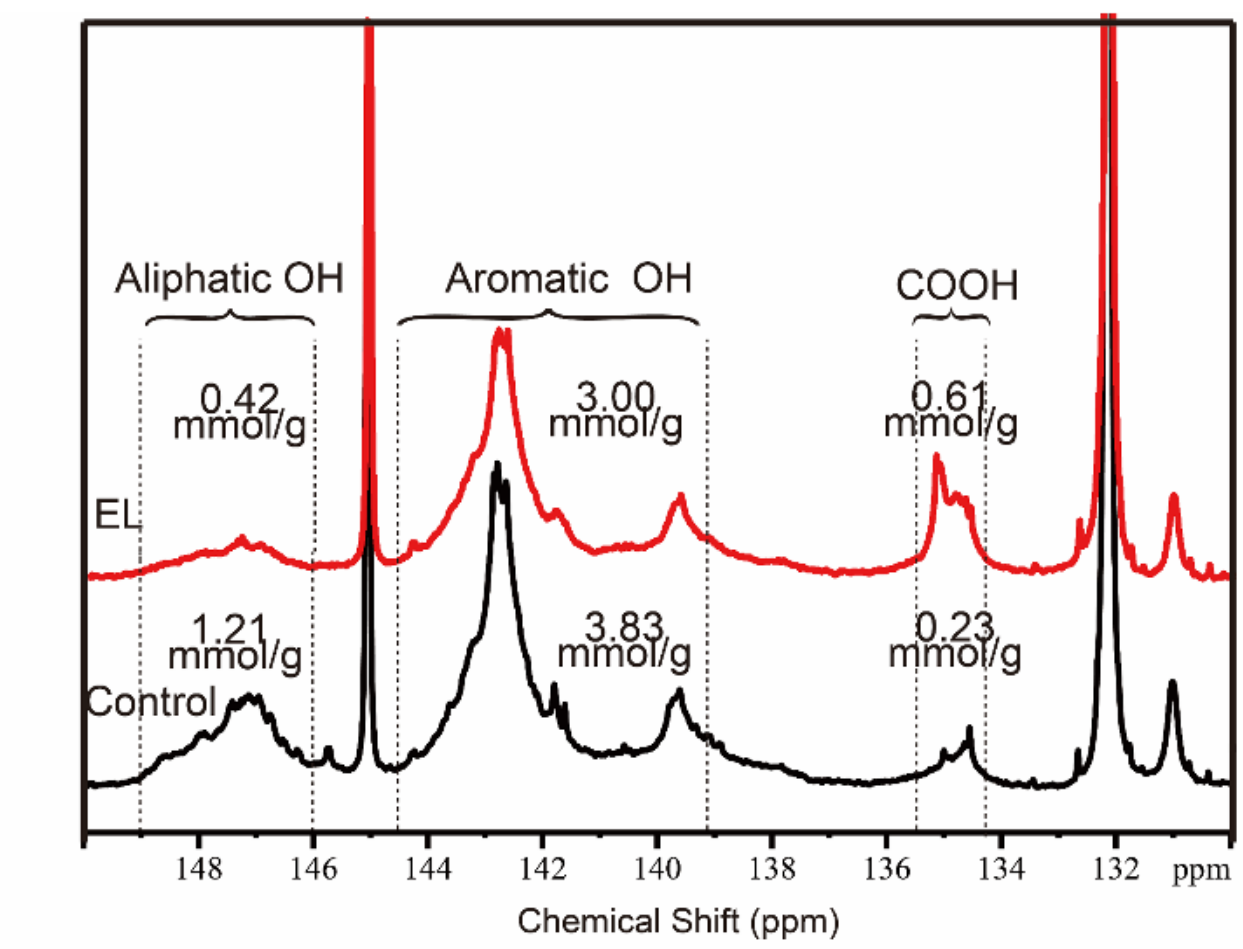

Figure S4. ^{31}P NMR of control and esterified lignin. ^{31}P NMR quantitative analysis revealed the obvious reduction in aliphatic and aromatic hydroxyl contents of EL, which verified the consumption of hydroxyl groups in esterification. The remarkably increased carboxyl content confirmed the successful introduction of reactive carboxyl groups via maleic anhydride grafting.

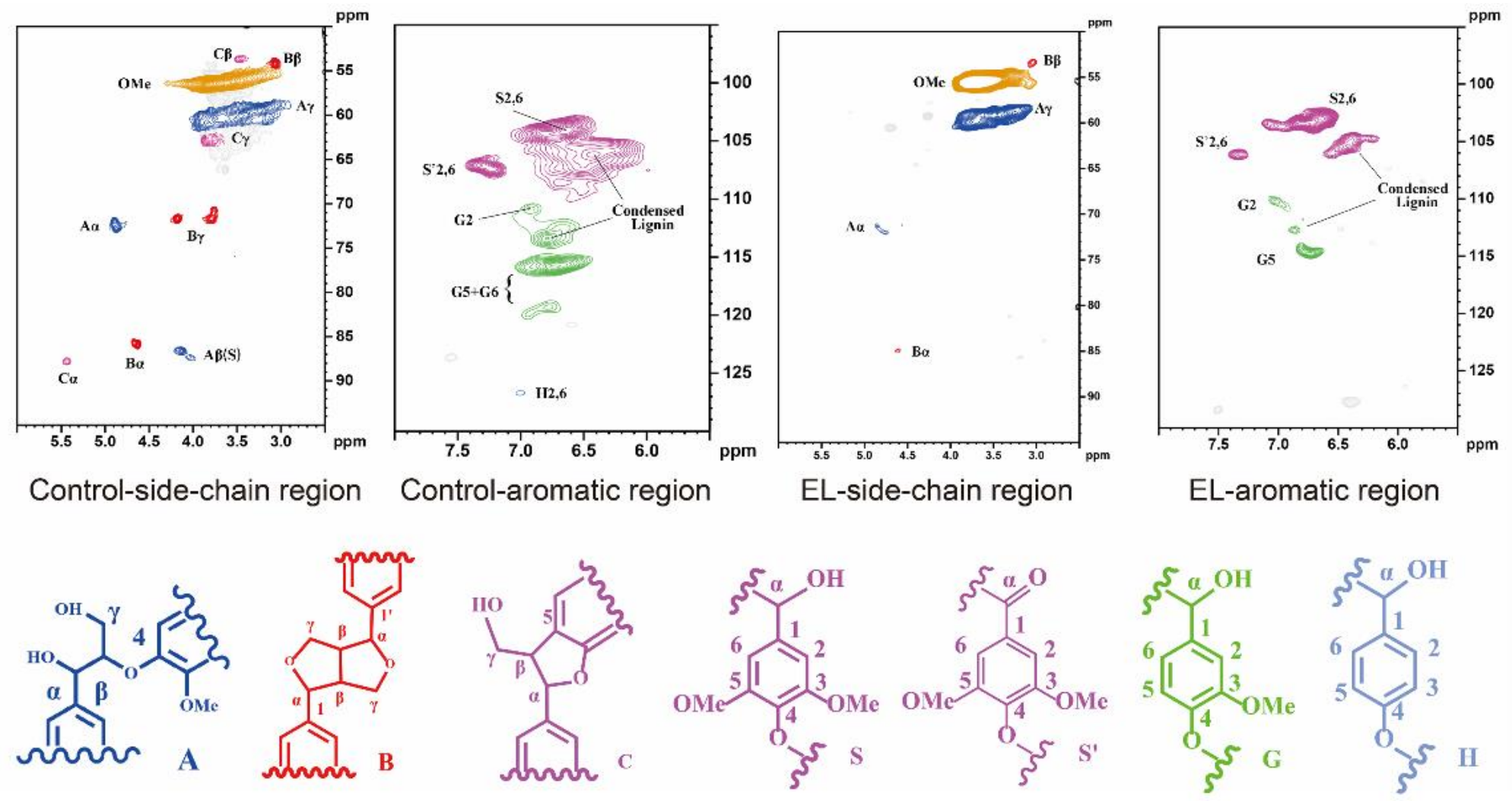

Figure S5. 2D-HSQC analysis of control and esterified lignin. 2D HSQC NMR analysis confirmed the successful grafting of maleic anhydride onto the lignin macromolecular structure. The esterification reaction altered the chemical environment as evidenced by the weakened or shifted characteristic correlation signals, indicating effective structural modification of lignin.

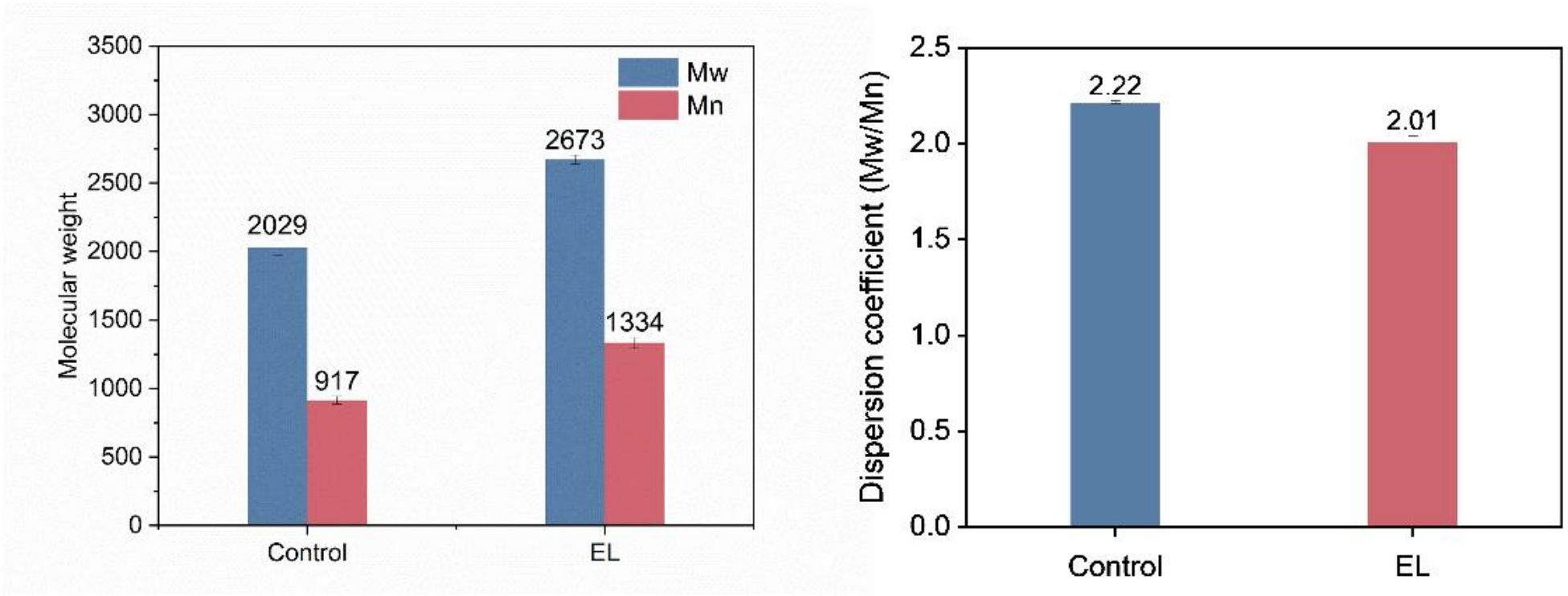

Figure S6. GPC analysis of control and esterified lignin.

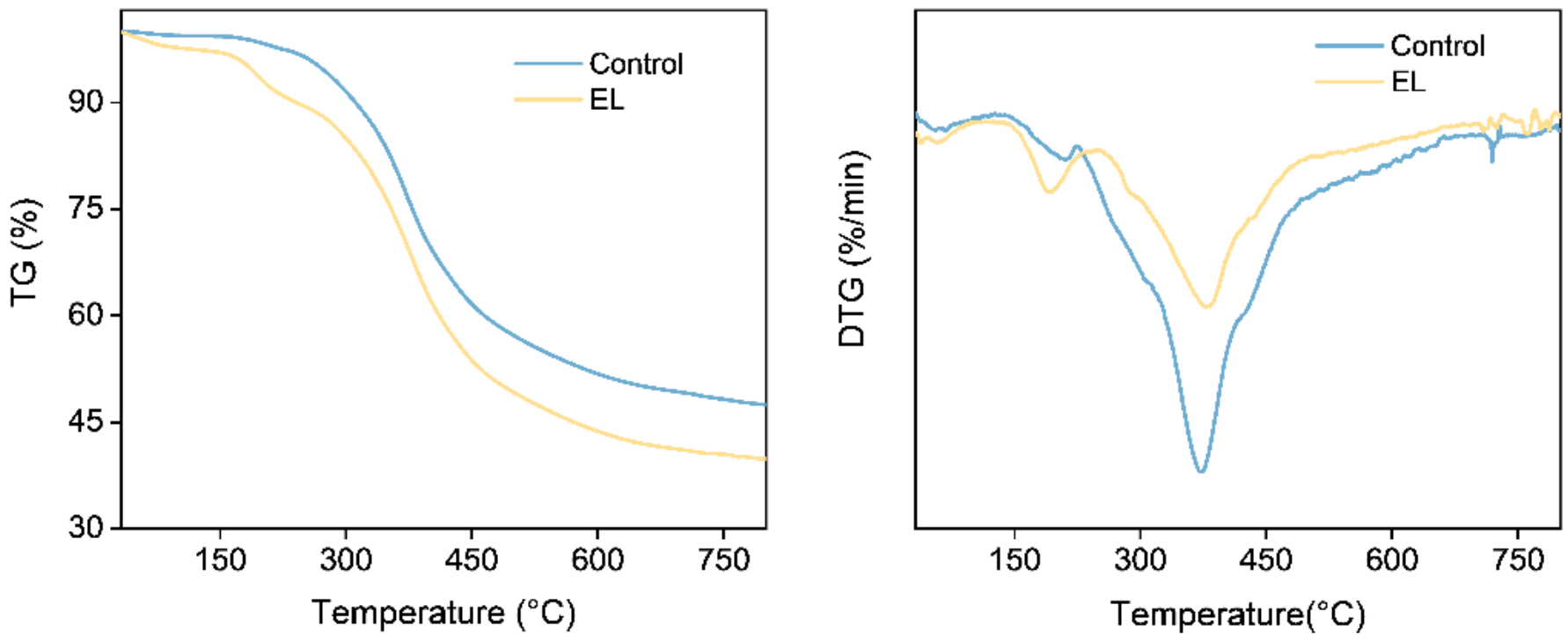

Figure S7. TG/DTG analysis of control lignin and esterified lignin. EL showed a lower maximum decomposition temperature and faster weight loss rate, demonstrating that the grafting modification reduced the thermal stability of lignin.

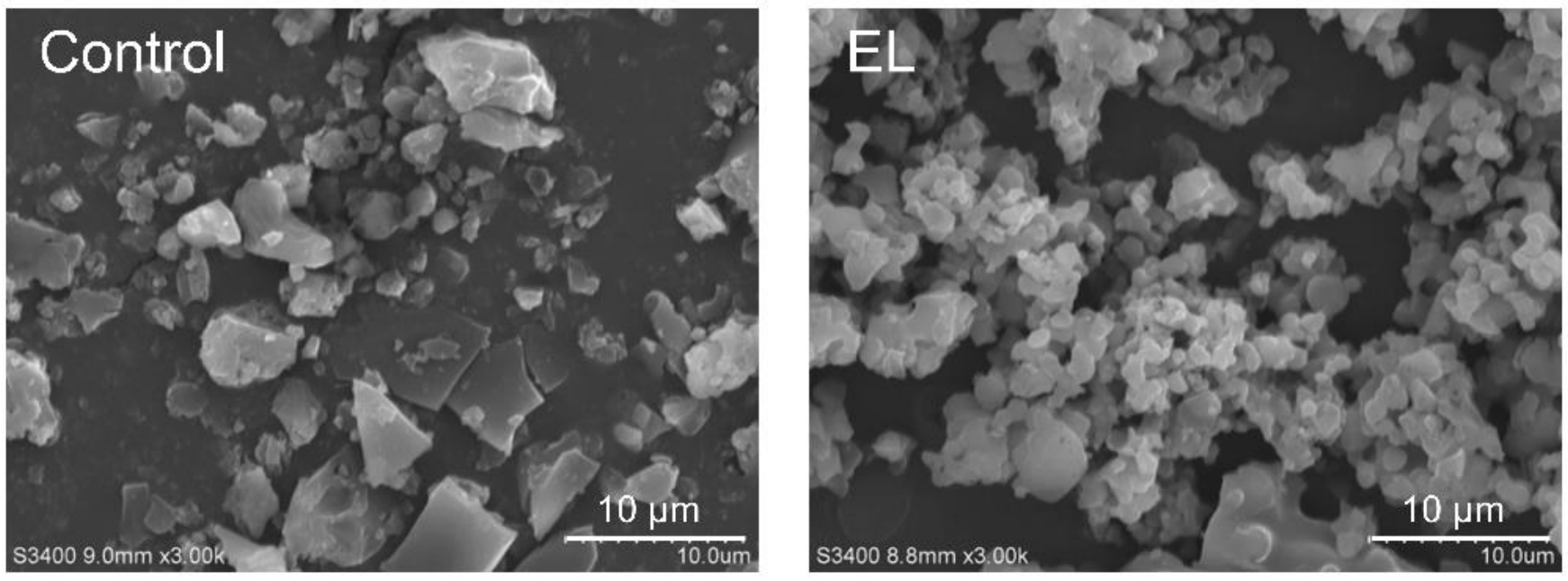

Figure S8. SEM images of control and esterified lignin.

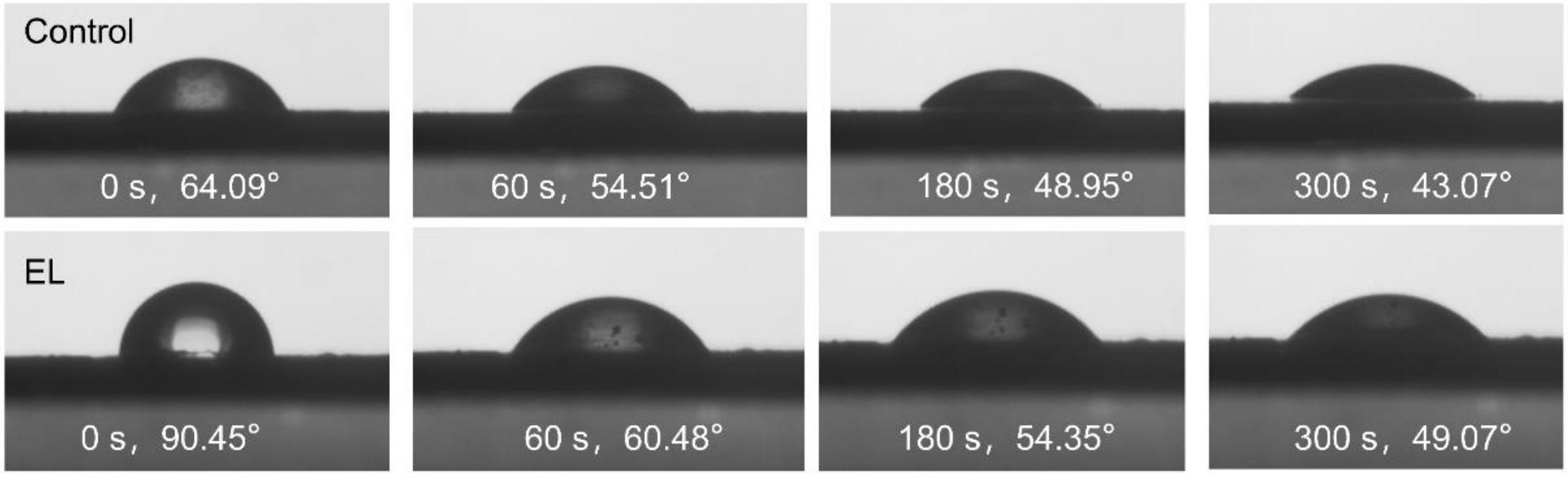

Figure S9. Water contact angle of control and EL.

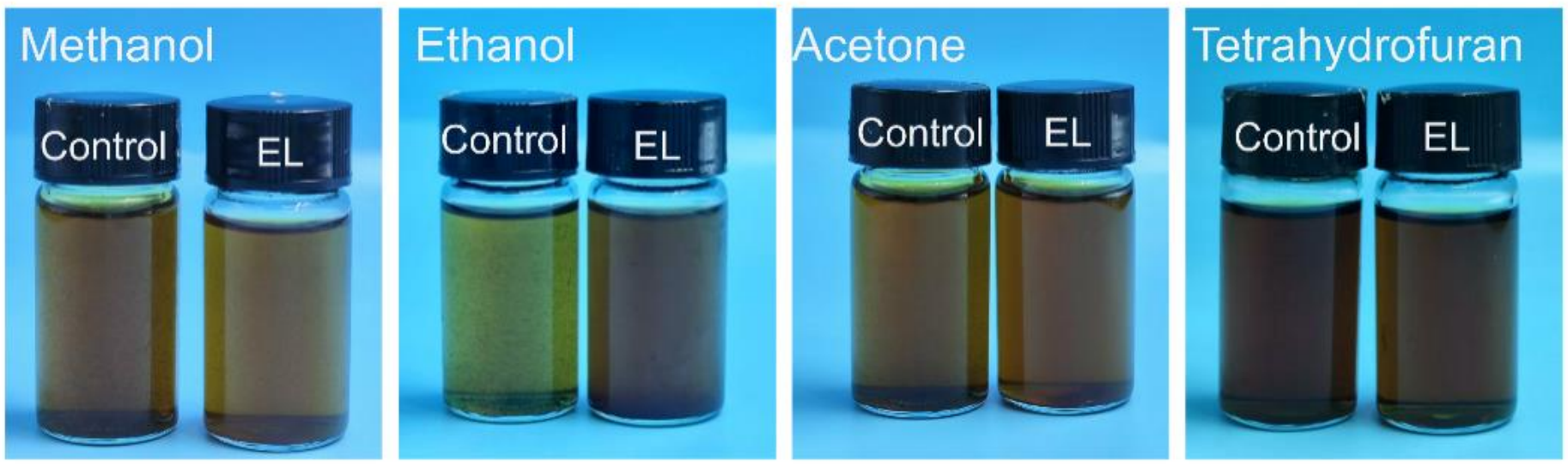

Figure S10. The solubility changes in maleic anhydride-modified lignin.

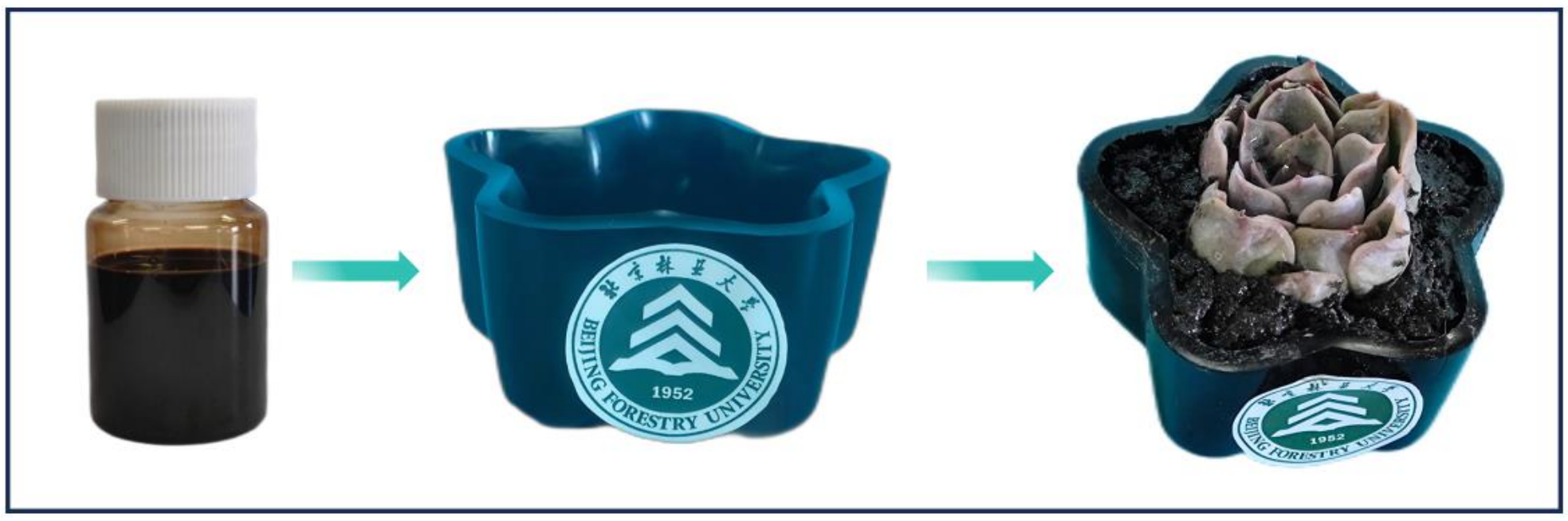

Figure S11 Thermal processability of the bio-based epoxy resin. The bio-epoxy resin was successfully thermo-molded into flower pots for succulent cultivation, confirming the favorable thermal processability of the modified lignin-based resin and its promising prospect in eco-friendly plastic applications.

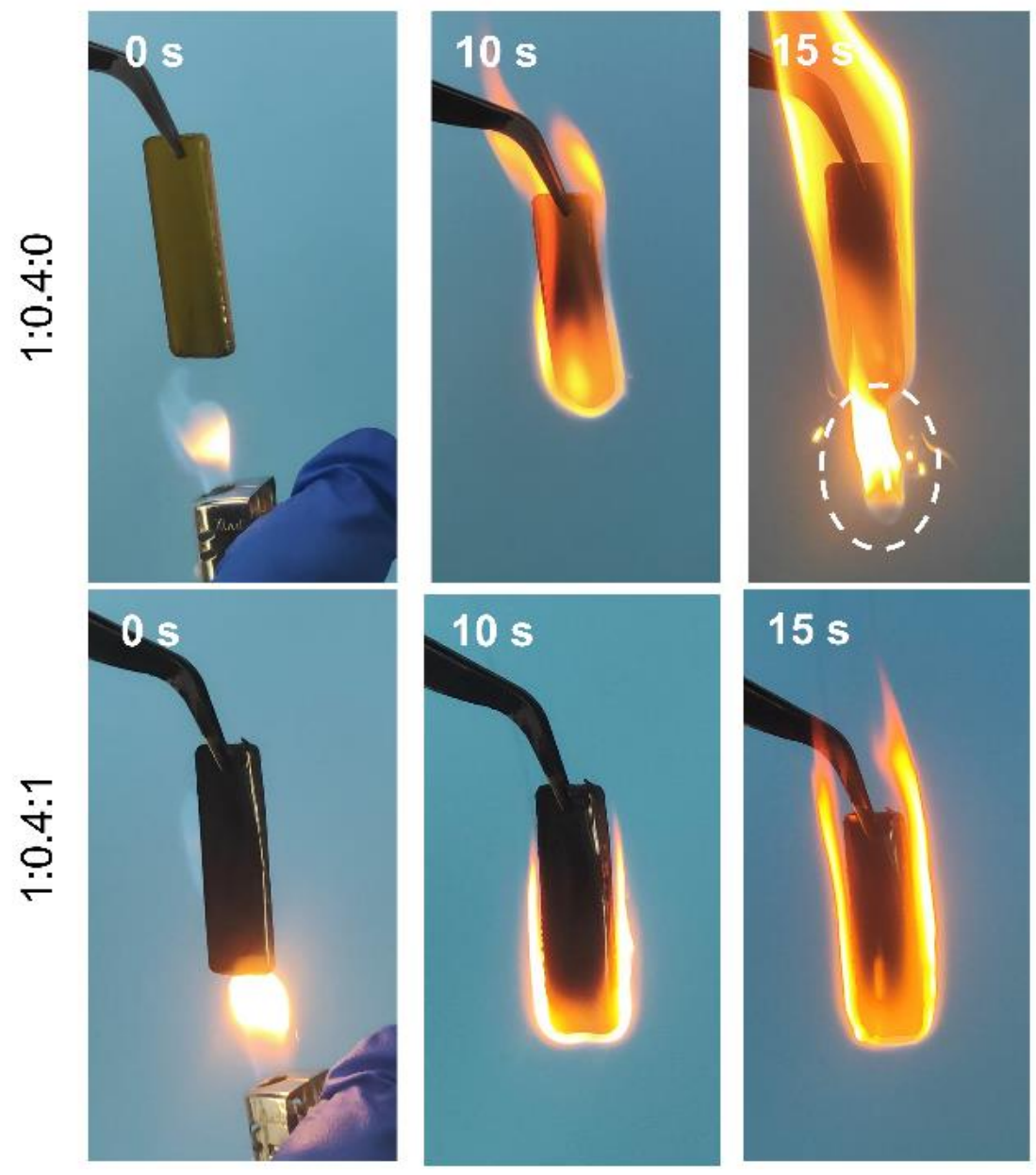

Figure S12. The vertical burning tests of bio-based epoxy resin.

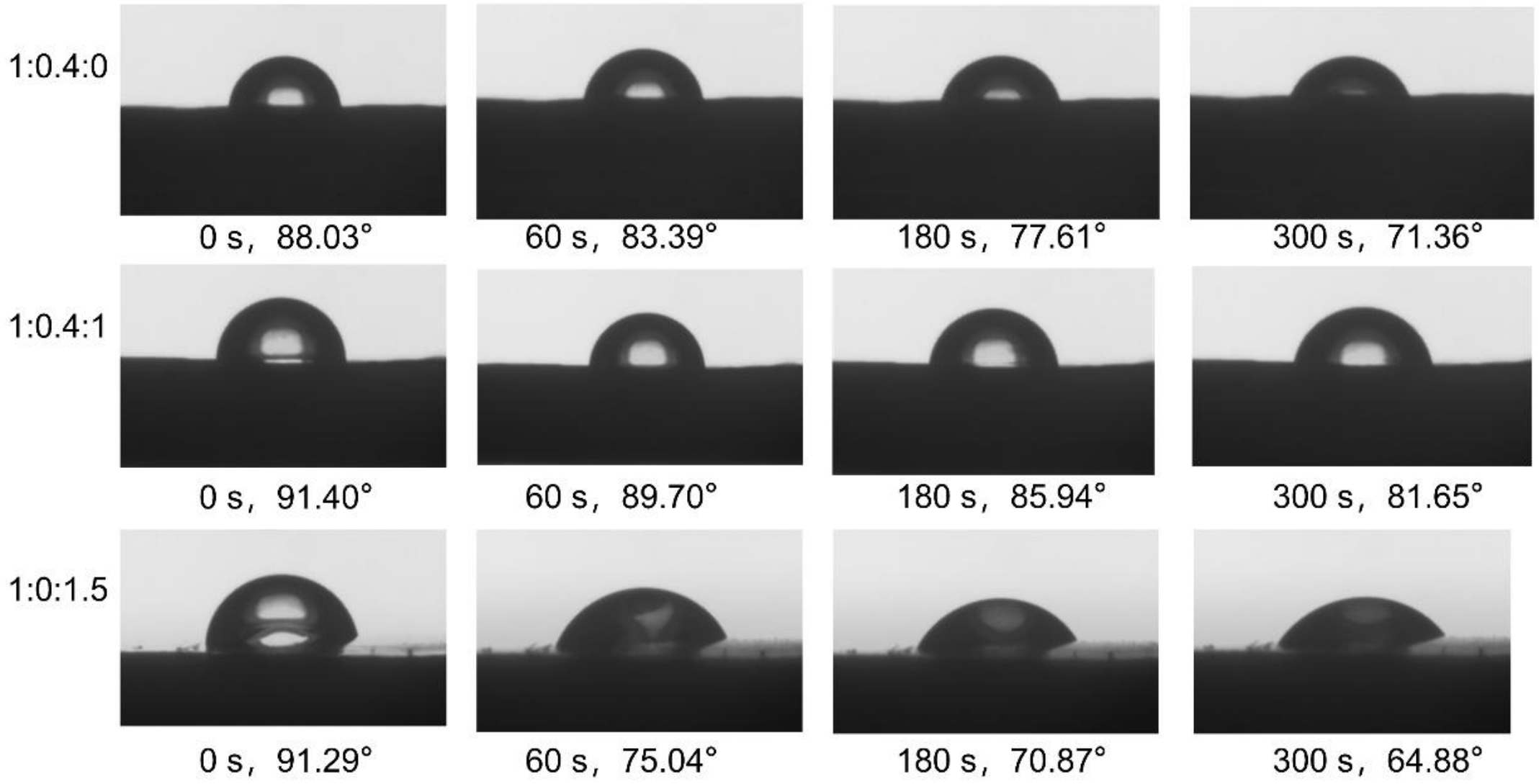

Figure S13. Water contact angle of bio-based epoxy resin.

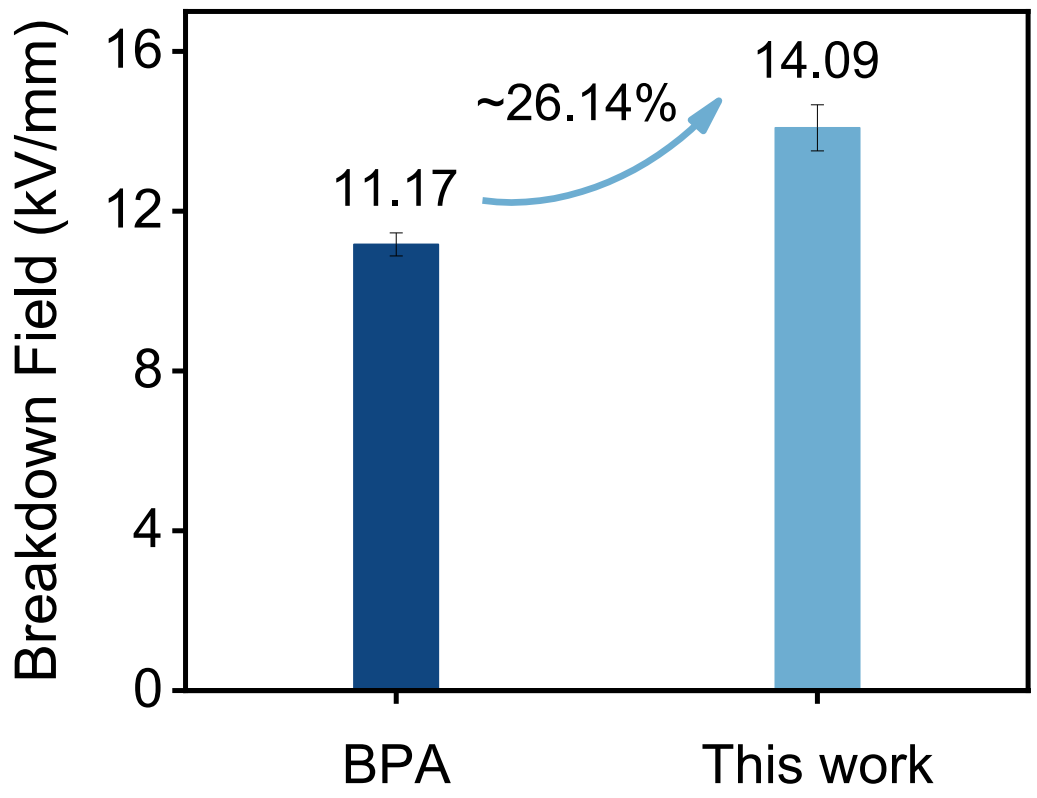

Figure S14 Breakdown strength of bio-based epoxy resins and BPA resin.

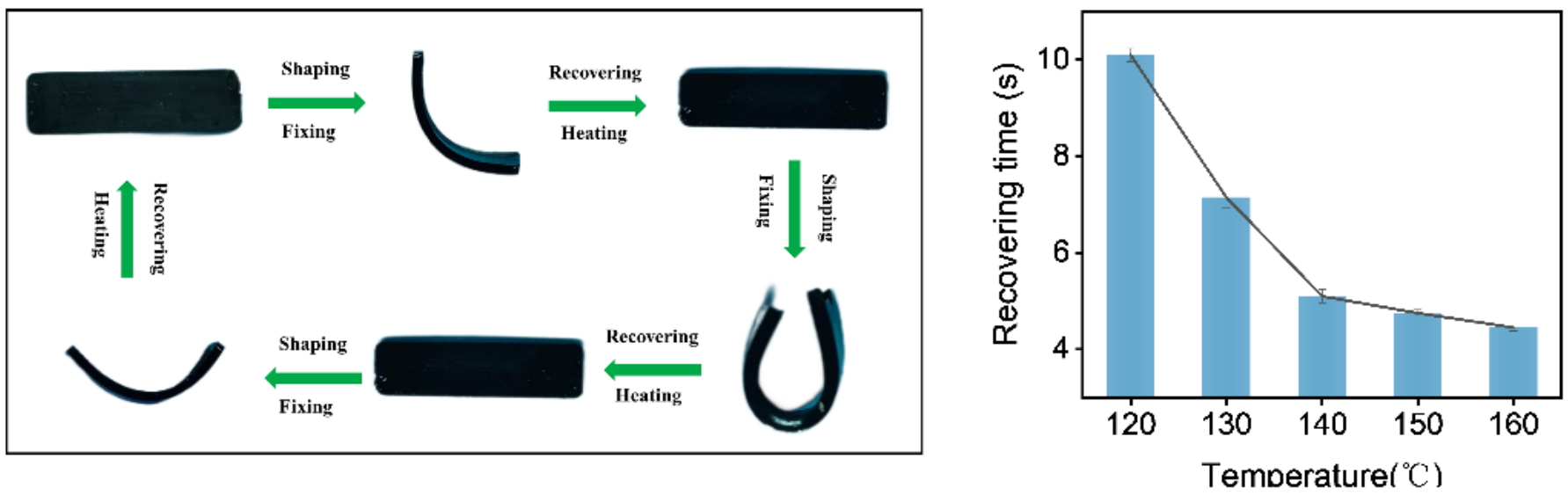

Figure S15 Shape memory behavior of bio-based epoxy resin and recovery time at different temperatures. The resin exhibited excellent repeatable thermally-responsive shape memory performance. The shape recovery time decreased continuously with rising temperature, owing to the accelerated movement of polymer chains under higher heating temperatures.

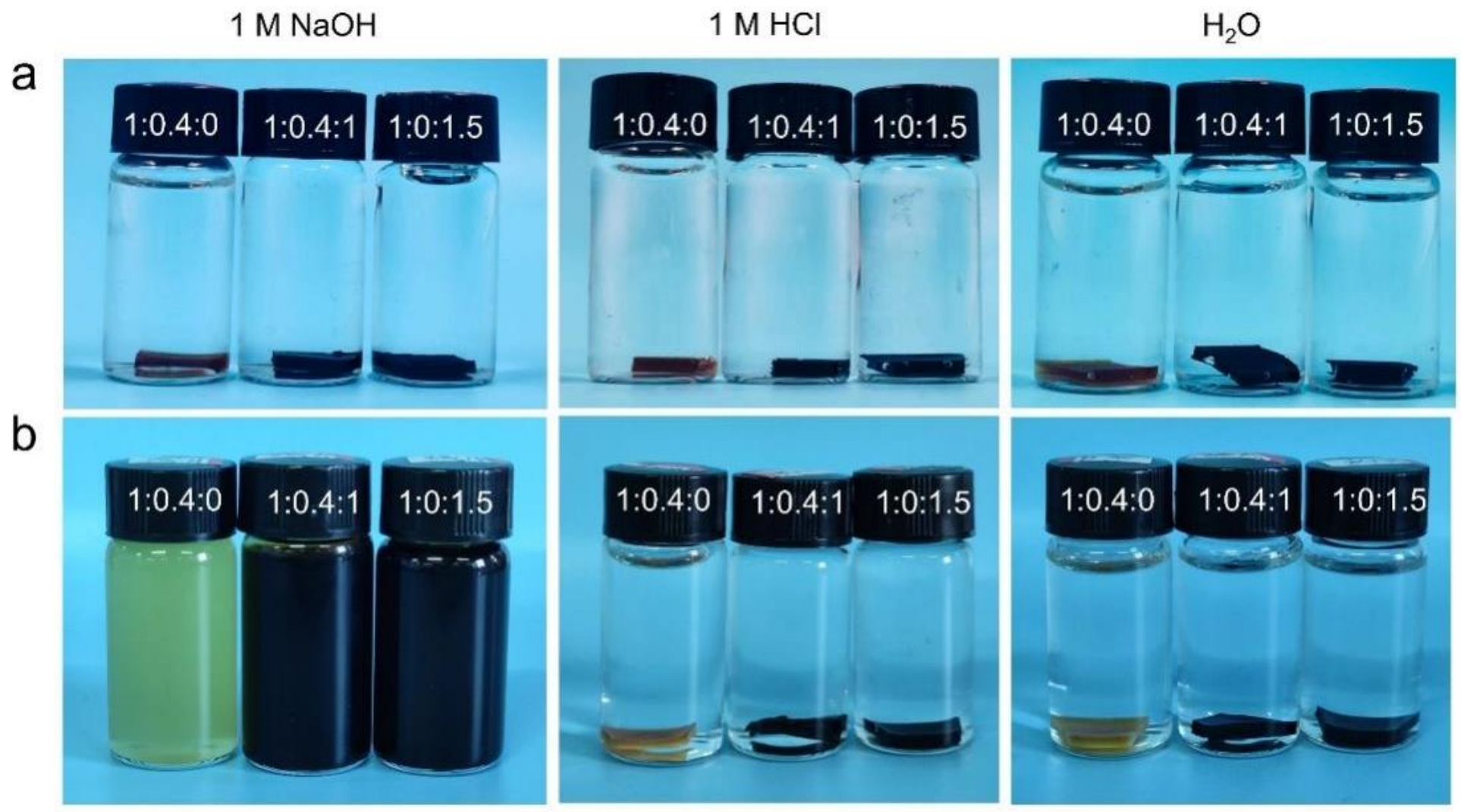

Figure S16 Digital images of bio-based epoxy resin after one week in different solvents.

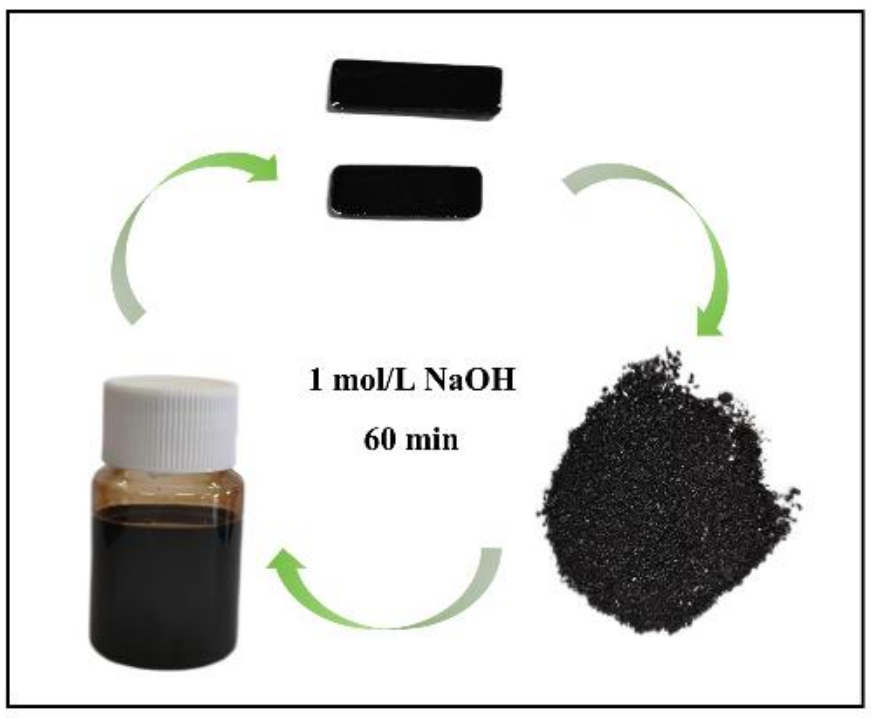

Figure S17 Recovery of bio-based epoxy resins by pH-responsive dynamic ester bond cleavage.

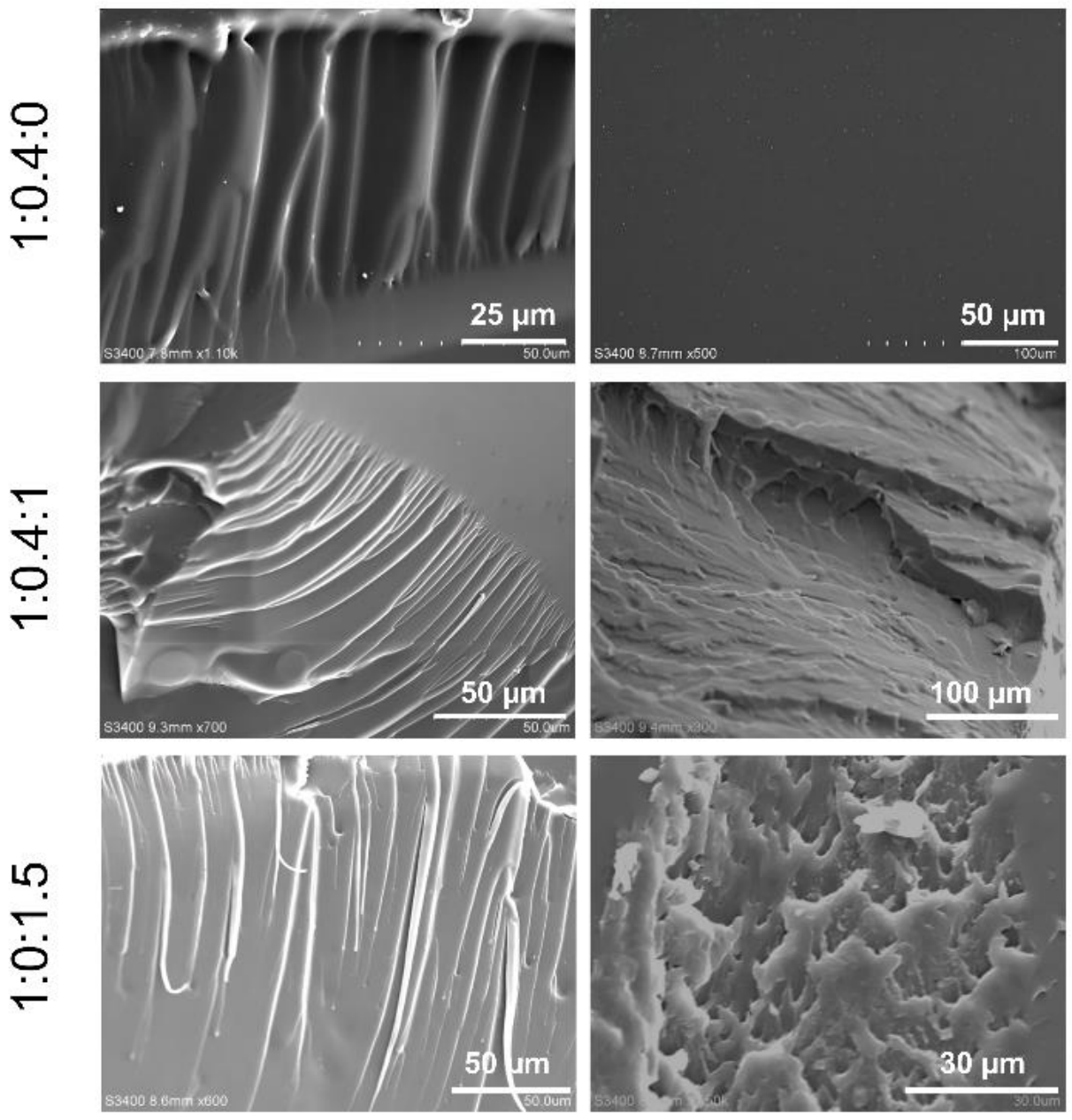

Figure S18 SEM of the liquid-nitrogen-fractured resins.

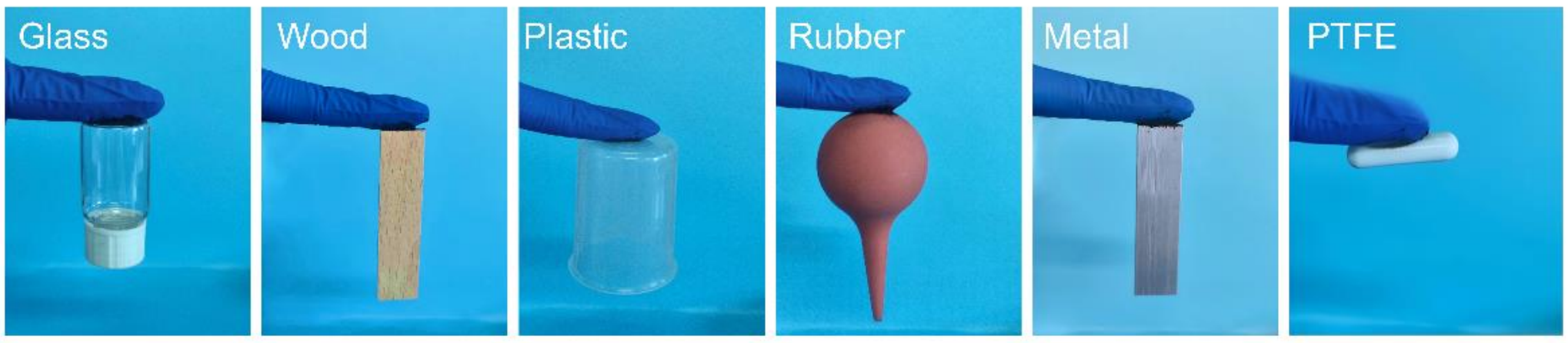

Figure S19 Bio-based epoxy adhesives tightly adhere to various substrates.

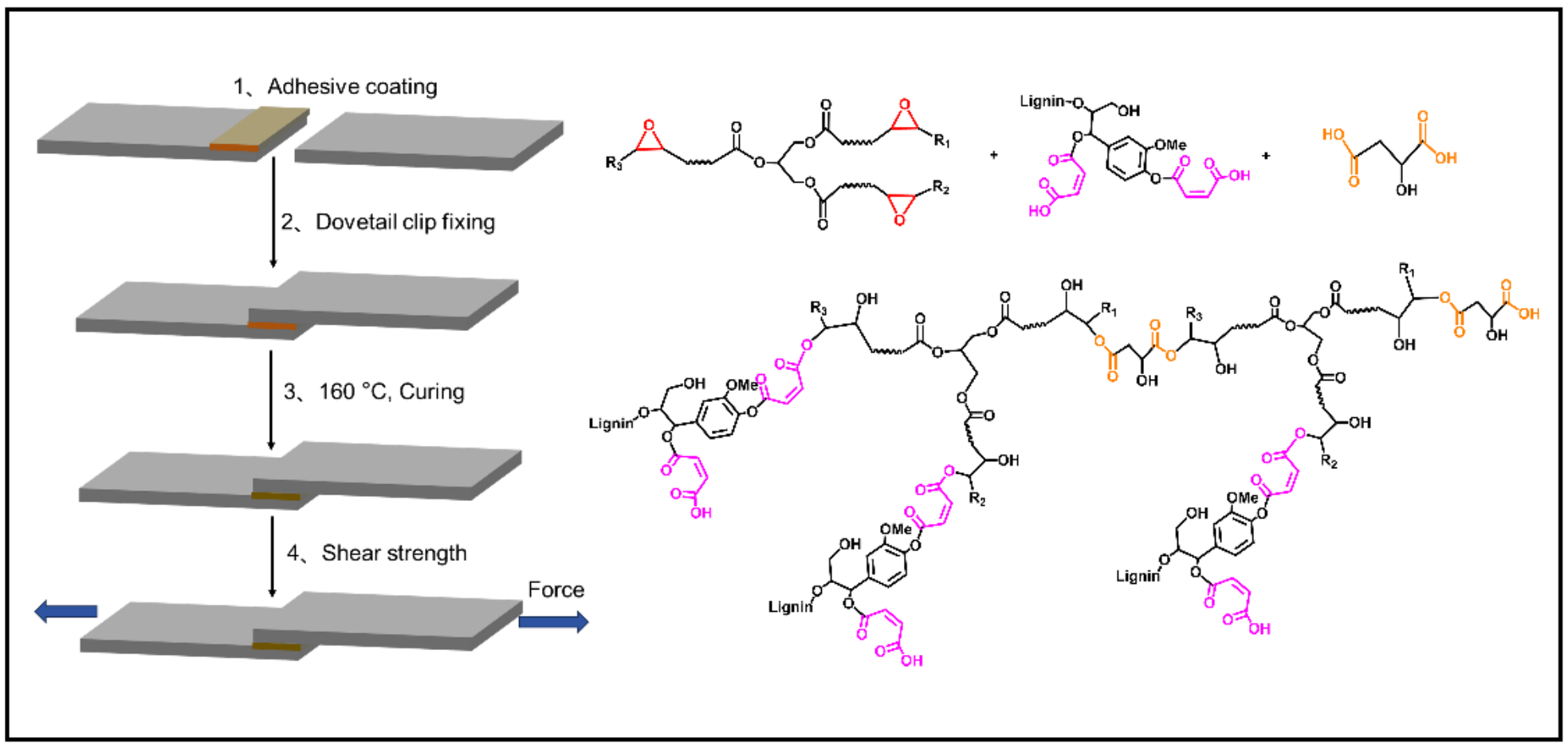

Figure S20 Schematic of experiments to measure lap shear adhesion and possible curing mechanisms for bio-based epoxy adhesives.

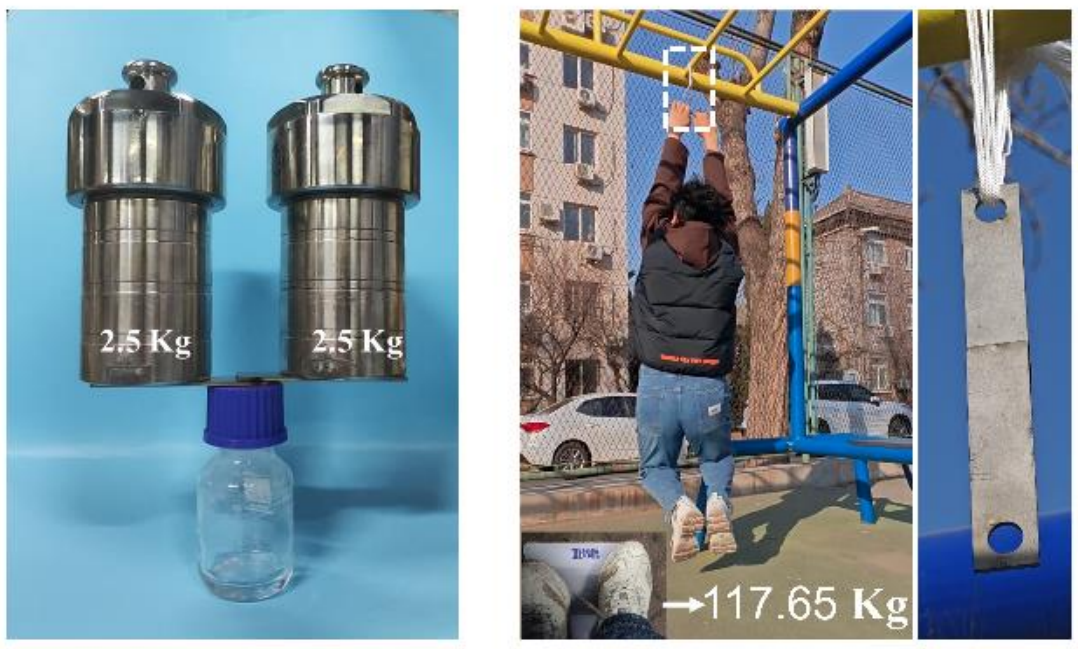

Figure S21 The shear strength was measured after the structure carried a 2.5 kg weight at each end and supported an approximately 120 kg adult male. (bonding area: 20 × 12 mm^2)

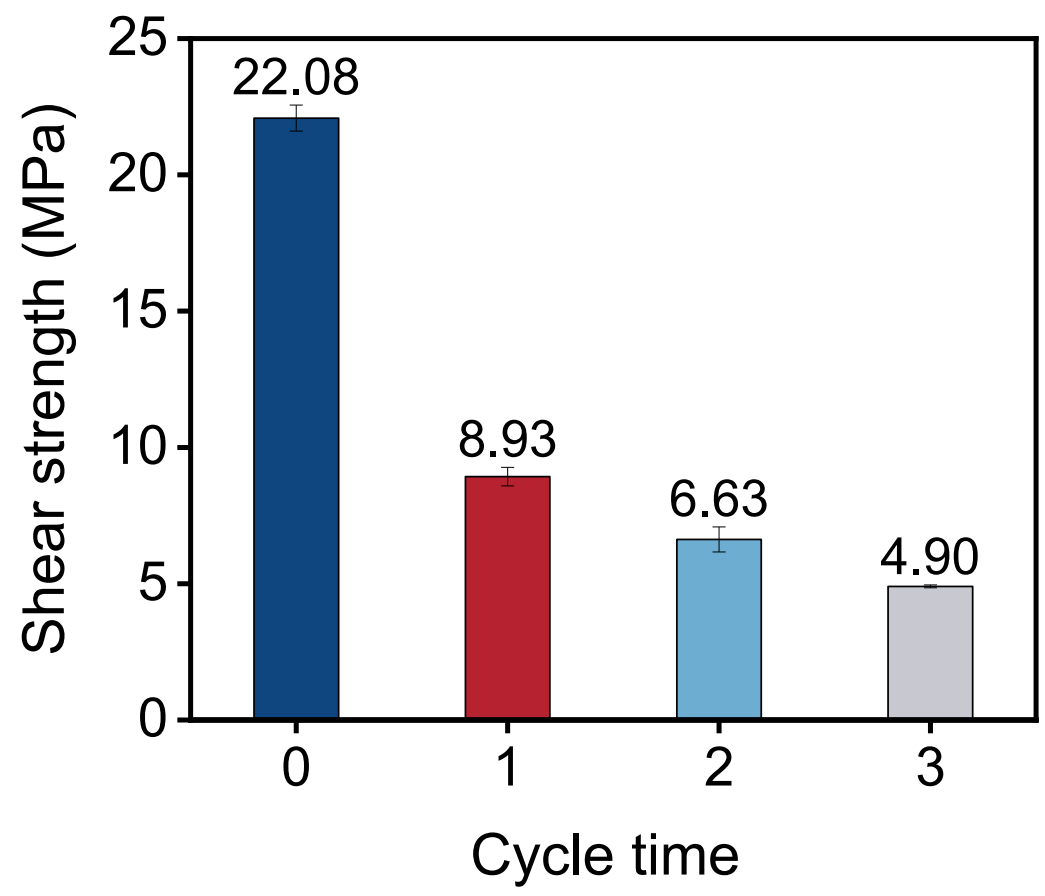

Figure S22 Shear strength of bio-based epoxy resin on stainless steel after different cycle time.

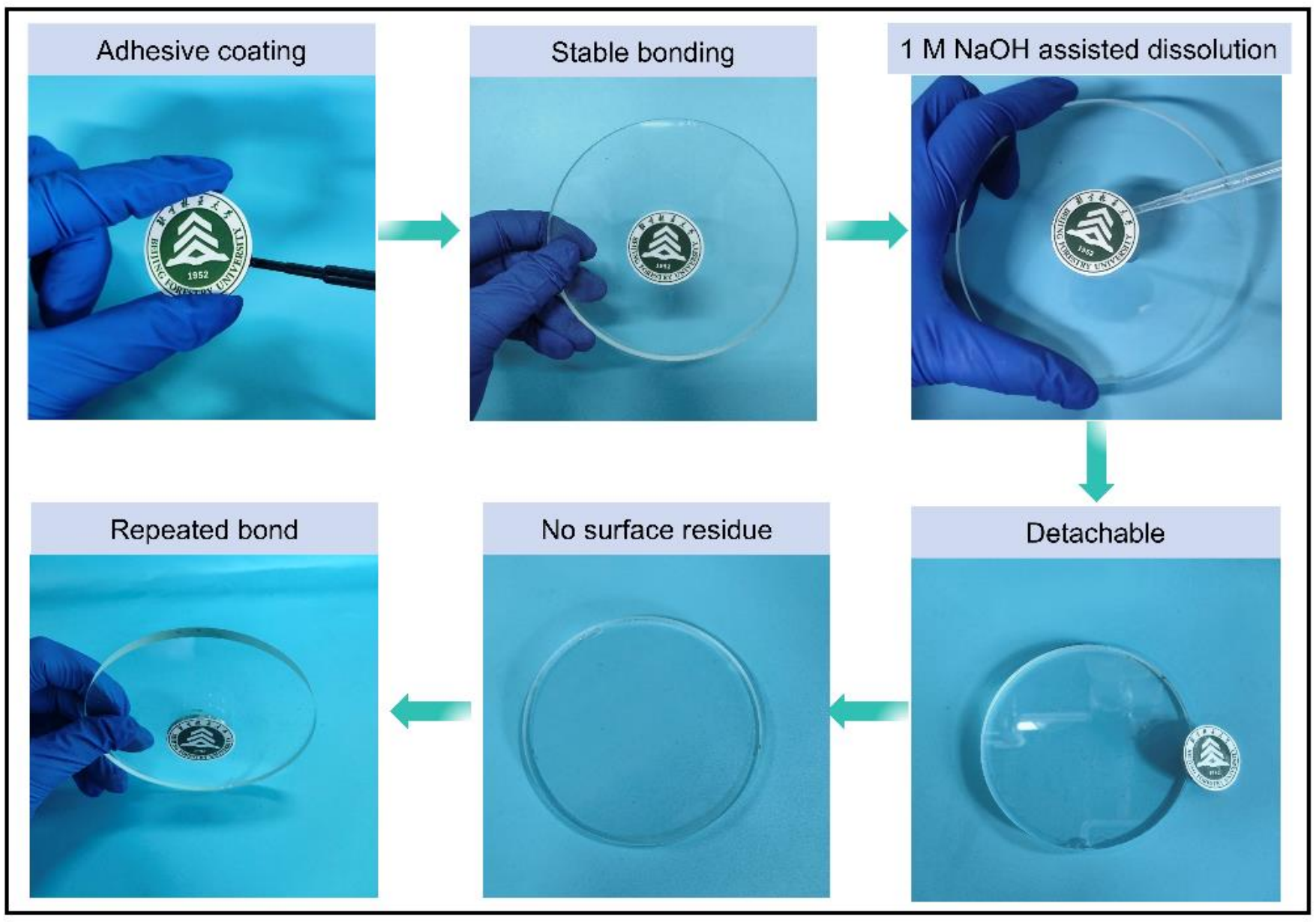

Figure S23 Demonstration of the bio-based epoxy adhesive for stable bonding of university-emblem stainless-steel sheets on glass substrates, followed by alkali-triggered residue-free detachment and repeatable re-bonding.

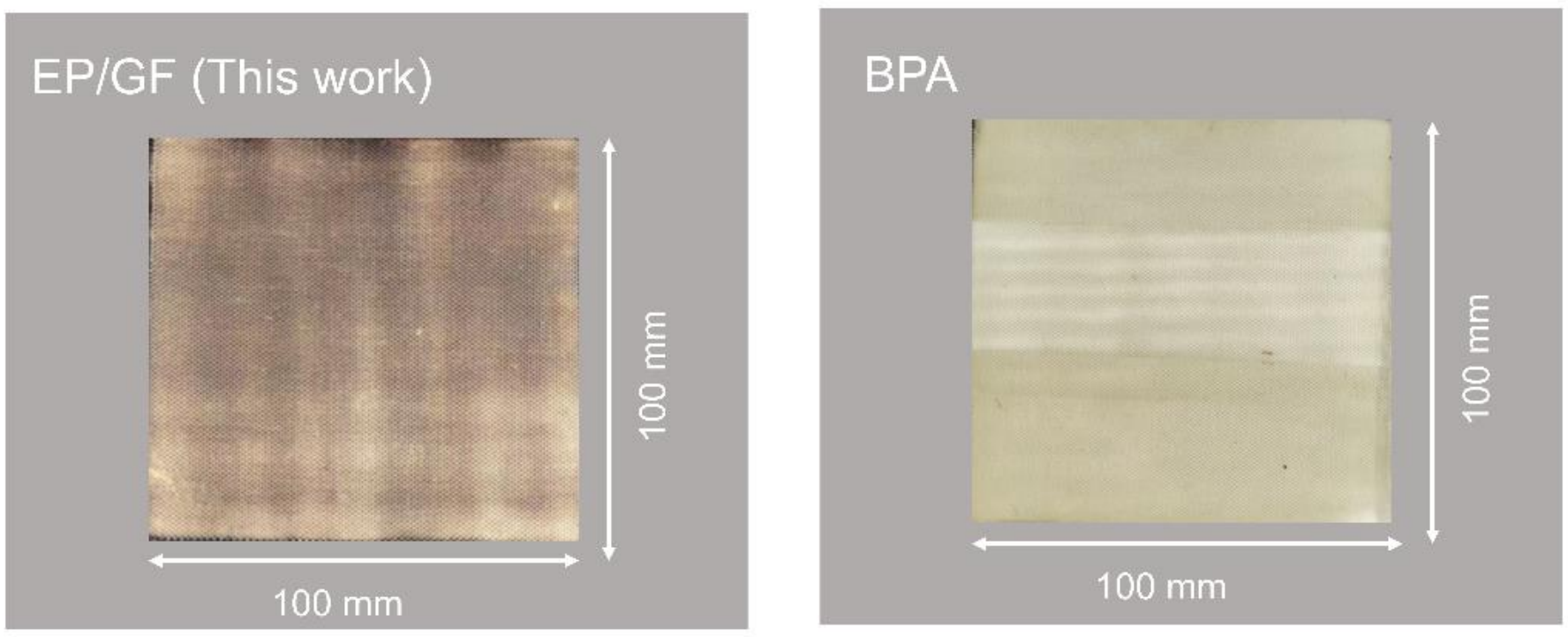

Figure S24 Digital images of epoxy resin/glass fabric composites.

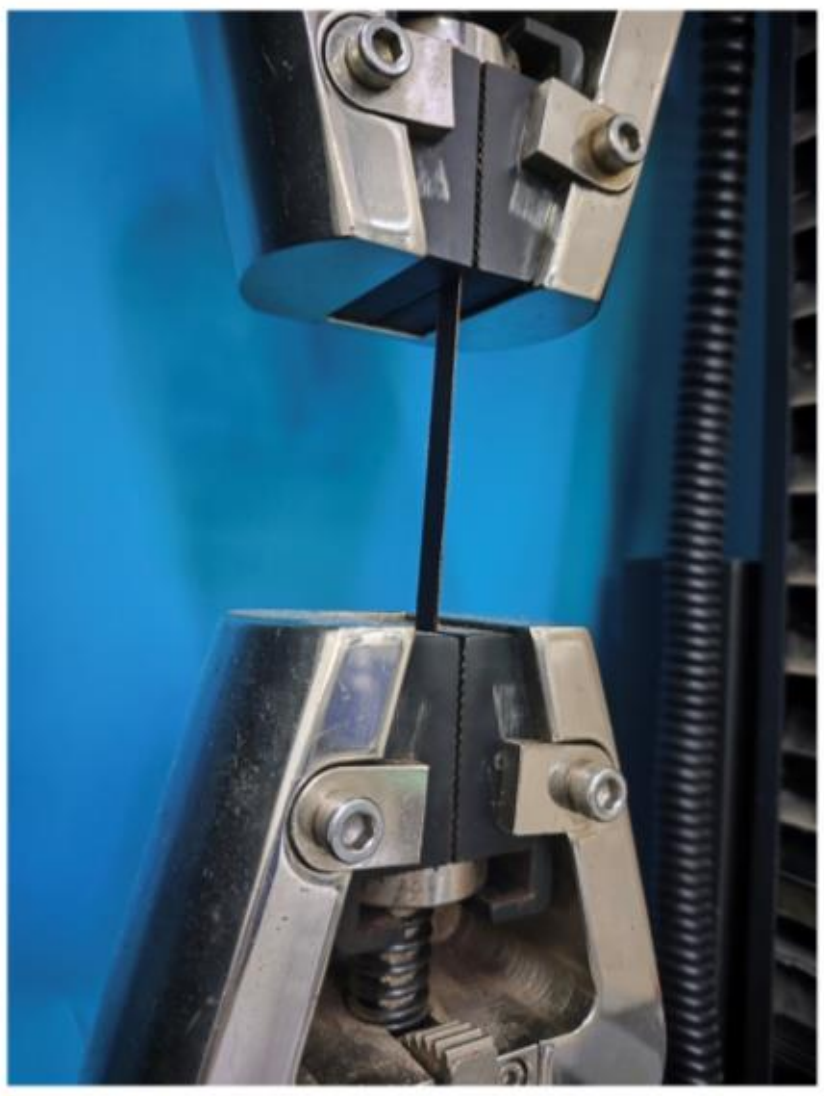

Figure S25 Schematic diagram of epoxy resin/glass fabric composites tensile strength test.

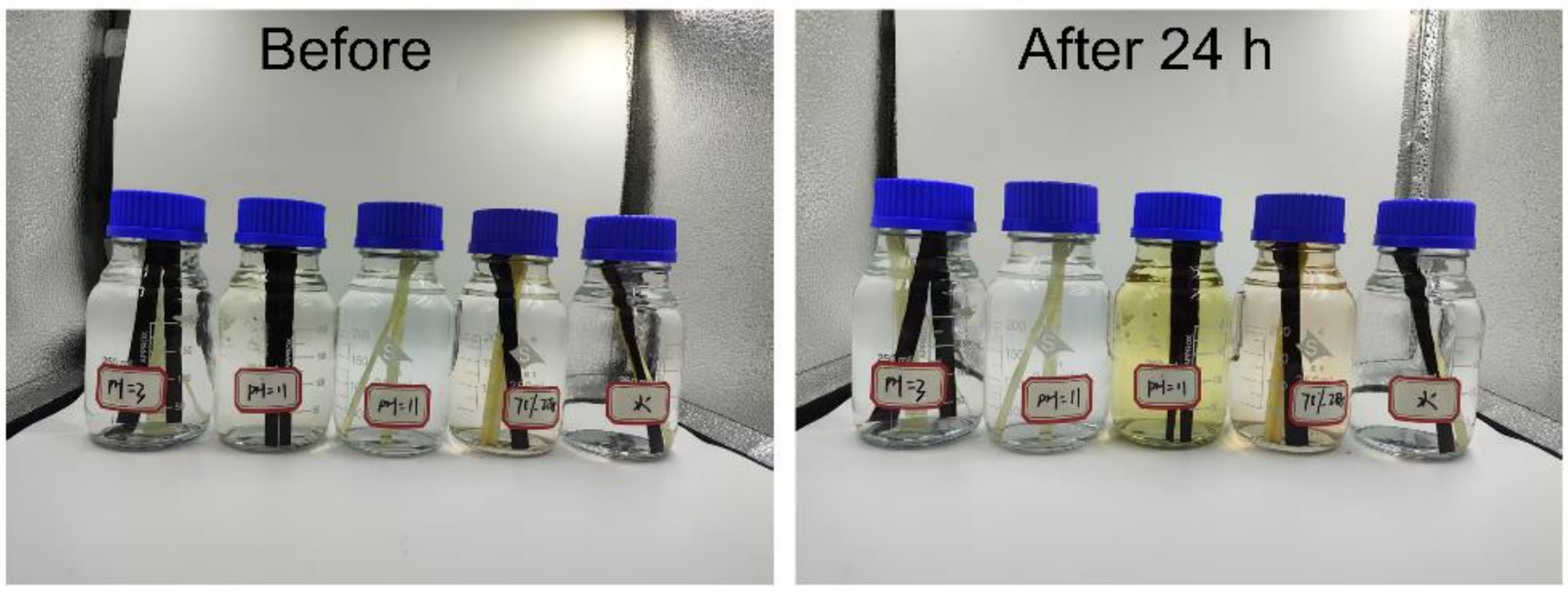

Figure S26 Digital images of epoxy resin/glass fabric composites immersed in acid, alkali, ethanol and water for 24 hours. The composite exhibited excellent stability in acid and water, whereas partial resin dissolution was observed in alkaline solution and ethanol, which confirmed its controllable degradability and recyclable property.

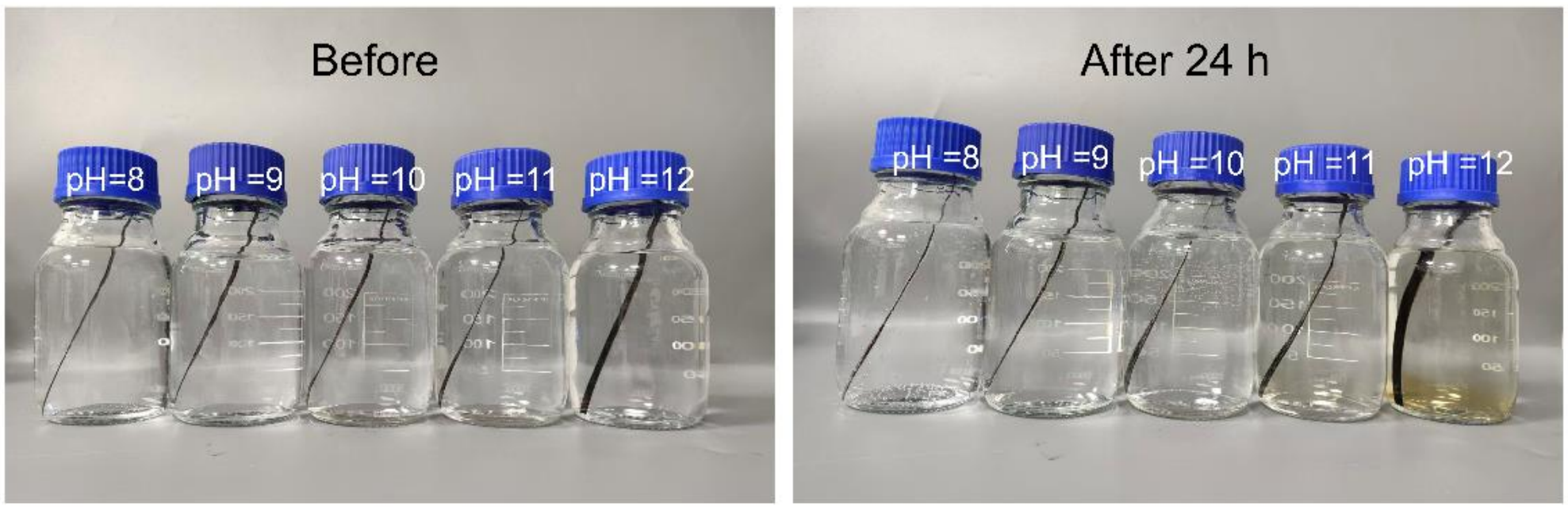

Figure S27 Digital images of epoxy resin/glass fabric composite materials after being immersed in different alkaline solutions for 24 hours. The composite exhibited favorable chemical stability in alkaline solutions with pH lower than 11, while obvious resin leaching occurred under strong alkaline condition (pH=12), which endowed the material with pH-responsive controllable degradability.

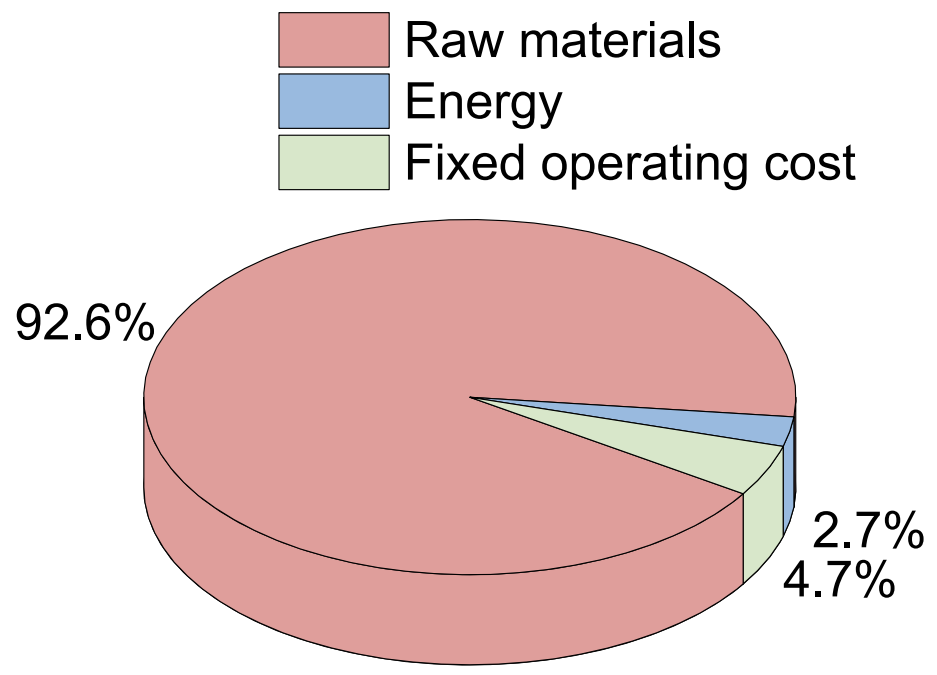

Figure S28 Percentage distribution of bio-based epoxy resins production plant operating costs.

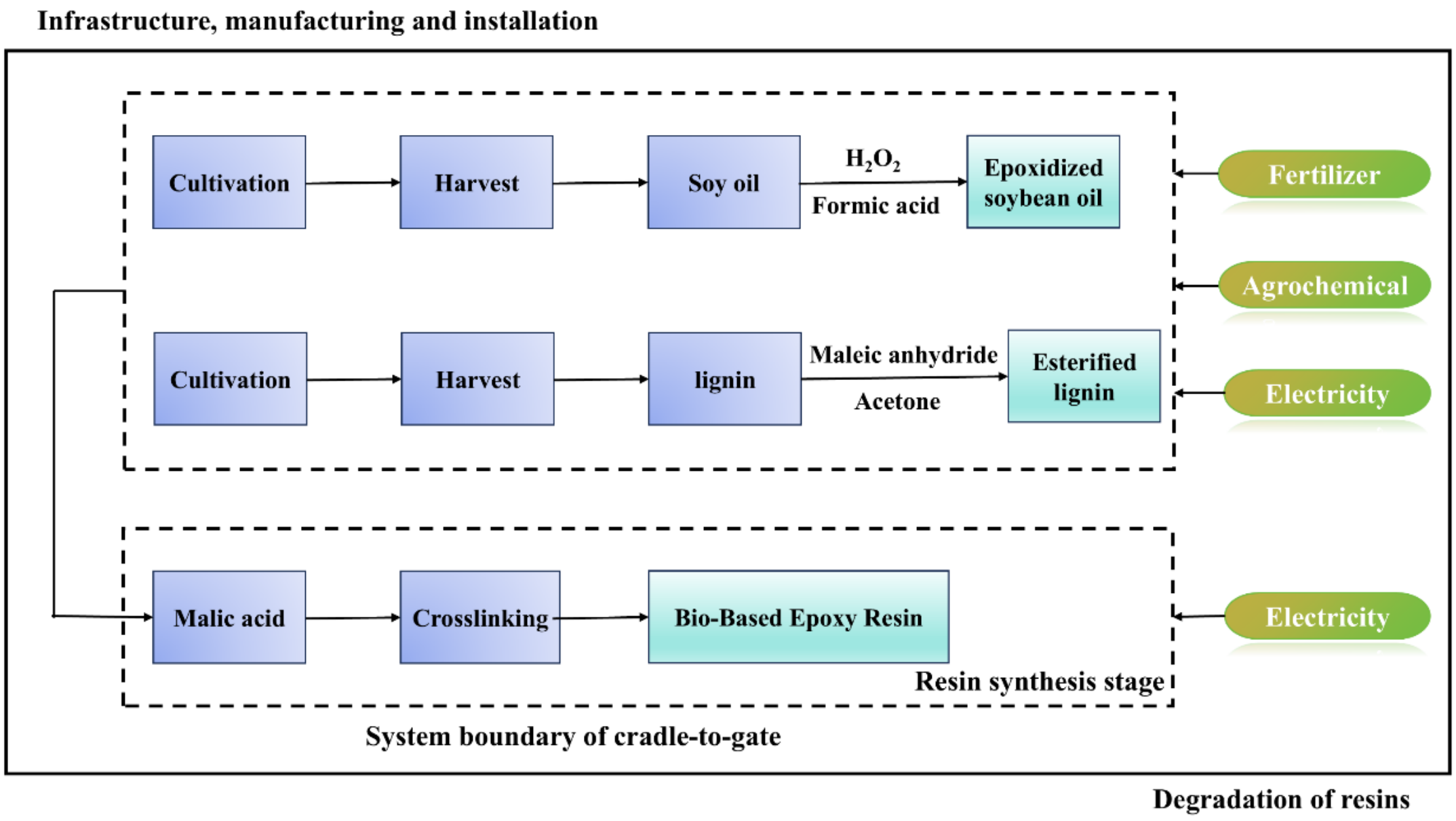

Figure S29 System boundary for bio-based epoxy resin production. The system boundary covered the whole manufacturing chain including raw material cultivation and harvesting, chemical modification of soybean oil and lignin, as well as the crosslinking synthesis procedure of bio-based epoxy resin. Key input resources such as fertilizer, agrochemicals and electricity were included in the inventory analysis. The degradation process of the final epoxy product was excluded from the defined cradle-to-gate system boundary.

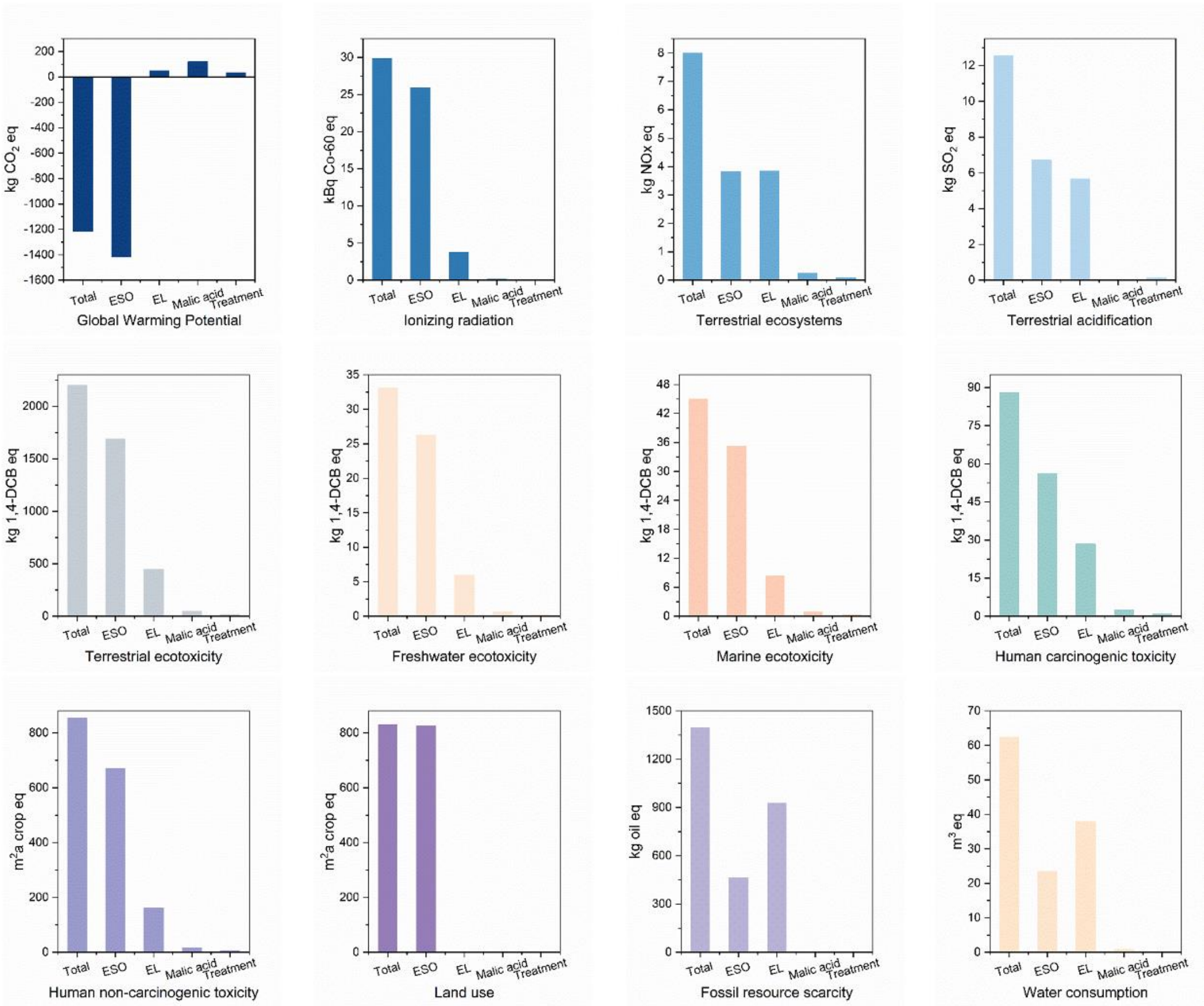

Figure S30 The main environmental impacts of the production of 1 ton bio-based epoxy resin.

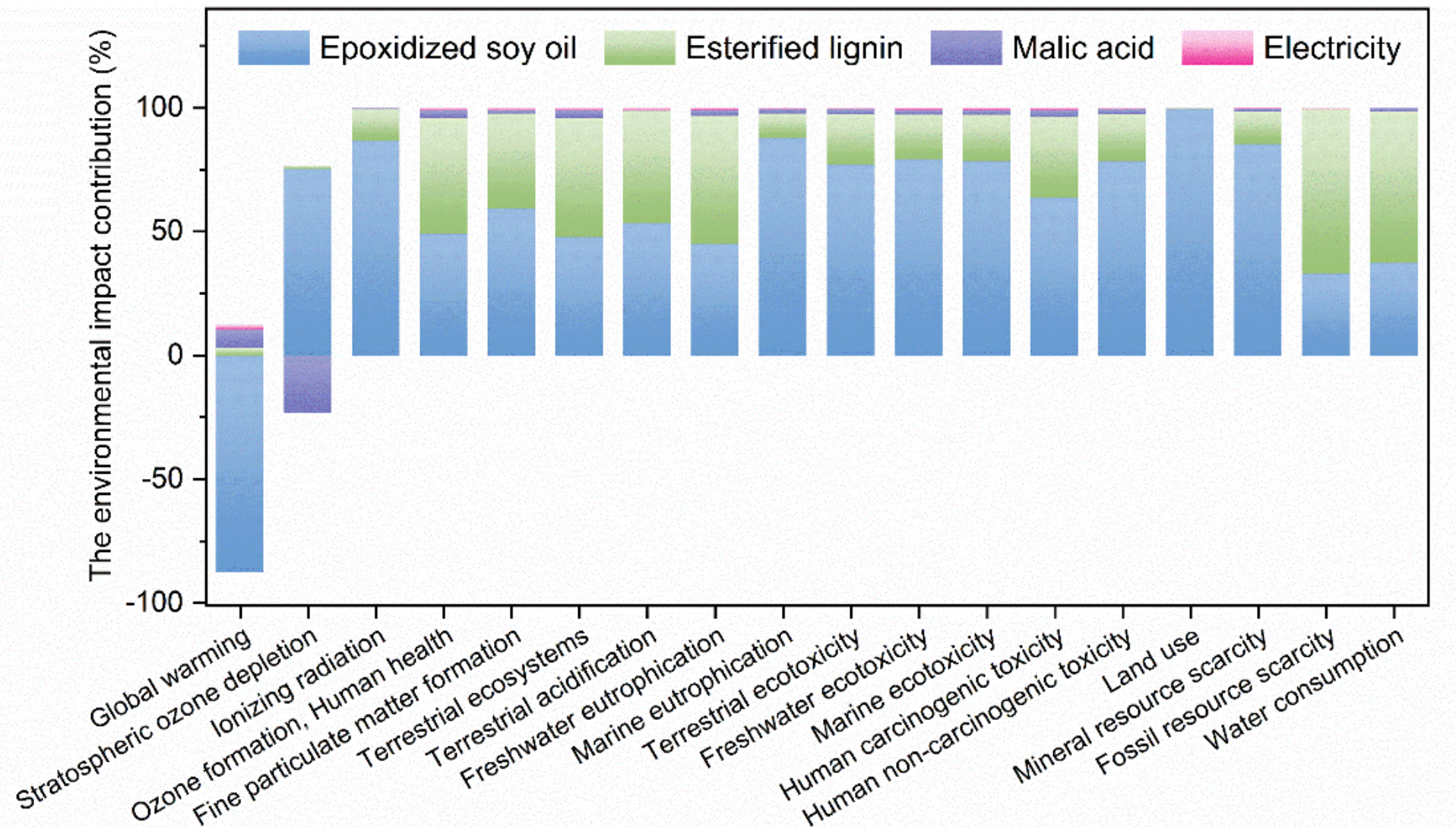

Figure S31 The environmental impact contribution (%) for all midpoint impact categories.

References

1. C. Zuo, X. Y. Hui, P. A. Xin, et al., "A green one-pot strategy for sustainable fully lignin-based adhesives," *Green chem* 28, (2026): 28. https://doi.org/10.1039/d5gc05305f

2. C. Moretti, B. Corona, R. Hoefnagels, et al., "Review of life cycle assessments of lignin and derived products: Lessons learned," *Sci Total Environ* 770, (2021): 144656. https://doi.org/10.1016/j.scitotenv.2020.144656

3. X. Lu and X. Gu, "A review on lignin-based epoxy resins: Lignin effects on their synthesis and properties," *Int J Biol Macromol* 229, (2023): 778-790. https://doi.org/10.1016/j.ijbiomac.2022.12.322
